# Development of a Synthetic 3-ketosteroid Δ^1^-dehydrogenase for the Generation of a Novel Catabolic Pathway Enabling Cholesterol Degradation in Human Cells

**DOI:** 10.1038/s41598-019-42046-8

**Published:** 2019-04-12

**Authors:** Brandon M. D’Arcy, Mark R. Swingle, Lindsay Schambeau, Lewis Pannell, Aishwarya Prakash, Richard E. Honkanen

**Affiliations:** 1 Department of Biochemistry & Molecular Biology, University of South Alabama, Mobile, AL 36688 USA; 2 Mitchell Cancer Institute, 1660 Springhill Ave, Mobile, AL 36604 USA

## Abstract

Cholesterol is an essential component of membranes, which is acquired by cells via receptor-mediated endocytosis of lipoproteins or via *de novo* synthesis. In specialized cells, anabolic enzymes metabolize cholesterol, generating steroid hormones or bile acids. However, surplus cholesterol cannot be catabolized due to the lack of enzymes capable of degrading the cholestane ring. The inability to degrade cholesterol becomes evident in the development and progression of cardiovascular disease, where the accumulation of cholesterol/cholesteryl-esters in macrophages can elicit a maladaptive immune response leading to the development and progression of atherosclerosis. The discovery of cholesterol catabolic pathways in *Actinomycetes* led us to the hypothesis that if enzymes enabling cholesterol catabolism could be genetically engineered and introduced into human cells, the atherosclerotic process may be prevented or reversed. Comparison of bacterial enzymes that degrade cholesterol to obtain carbon and generate energy with the action of human enzymes revealed that humans lack a 3-ketosteroid Δ^1^-dehydrogenase (Δ^1^-KstD), which catalyzes the C-1 and C-2 desaturation of ring A. Here we describe the construction, heterologous expression, and actions of a synthetic humanized Δ^1^-KstD expressed in Hep3B and U-937 cells, providing proof that one of three key enzymes required for cholesterol ring opening can be functionally expressed in human cells.

## Introduction

The causes of coronary vascular disease (CVD) are numerous^1^. Inherited defects in different aspects of lipoprotein metabolism, poor diet, a sedentary lifestyle, and secondary effects of other disorders (e.g. diabetes, hypothyroidism, and kidney disease), all contribute to disease^2–12^. Still, at a basic level, CVD is a disease of the intima. Atherosclerotic lesions start with endothelial damage or dysfunction in the arteries, allowing the accumulation of lipoproteins (principally low density lipoproteins; LDLs) in the intima^13^. To clear the intima of lipoproteins and lipoprotein debris, monocytes infiltrate the subendothelial space and differentiate into macrophages^14,15^. Macrophages ingest the cholesterol-rich lipoproteins via LDL- and scavenger-receptor mediated endocytosis^16,17^. To combat the cytotoxicity associated with a buildup of free intracellular cholesterol, acyl-CoA-acyltransferase (ACAT; SOAT1) converts excess cholesterol into cholesteryl esters (CE)^18–20^. CEs are relatively inert and accumulate as cytoplasmic lipid inclusions. High intracellular cholesterol also induces the expression of ATP-binding cassette-transport proteins (ABC-transporters), which aid in cholesterol efflux from macrophages to passing Apo-A1, HDLs, and possibly other lipoprotein particles^21–23^. In addition, high intracellular cholesterol triggers the suppression of HMG-CoA reductase activity (preventing cholesterol synthesis), the suppression of LDL-receptor expression, and the degradation of existing LDL-receptors^24–27^. In most cells, these feedback mechanisms are sufficient to maintain normal cholesterol homeostasis. However, scavenger receptor-mediated mechanisms (e.g. SR-AI/AII, CD36, CD68, LOX1) that take in LDLs are not suppressed by sterols^28,29^. Thus, when uptake exceeds efflux, the macrophages become engorged with CEs generating cells with a “foamy” appearance^20,29,30^. Foam cell formation helps trigger a complex maladaptive inflammatory response, leading to the development and progression of atherosclerosis and CVD^31^. Therefore, at a fundamental biochemical level the inability of macrophages to degrade surplus cholesterol is an important aspect of both the initiation and progression of CVD.

Although high serum cholesterol is associated with CVD, cholesterol is also an important component of cell membranes. Most cells can synthesize cholesterol if needed. However, the majority of body cholesterol is either acquired from the diet or generated via *de novo* synthesis in the liver. The liver converts excess cholesterol into cholesterol esters (CEs). Both cholesterol and CEs are exported from the liver in triglyceride rich, very low-density lipoproteins (VLDLs). During normal fat metabolism, the VLDLs give up their triglycerides to adipocytes for storage as fat, generating cholesterol/CE rich LDLs. Cells in need of cholesterol can express cell surface LDL-receptors, which take in the cholesterol rich LDLs. In the liver, cholesterol is also metabolized to generate bile acids, which are secreted into the intestinal lumen to aid fat absorption^32^. Due to efficient uptake mechanisms in the intestine, most bile acids are reabsorbed, and only small amounts are lost in the feces^33^. Nonetheless, when hepatic cholesterol levels are insufficient to meet this metabolic need, the expression of LDL-receptors is induced. This allows the liver to “recycle” cholesterol from the blood via the endocytosis of circulating LDLs. Drugs, collectively referred to as statins, exploit this biological process. Statins inhibit HMG-CoA reductase activity, and in doing so, suppress hepatic cholesterol synthesis^34,35^. In turn, the expression of LDL-receptors is induced, and serum lipoproteins are endocytosed to provide cholesterol for the metabolic needs of the liver. Statins, along with the recently developed PCSK-9 inhibitors (which prevent LDL-receptor degradation) have become the mainstays for lowering the circulating levels of LDLs in the blood.

To determine the molecular basis for the inability of human cells to degrade surplus cholesterol, we extensively examined studies of sterol metabolism. In addition to ACATs, human cells express CE-esterases, which convert cholesterol esters back to free cholesterol^36,37^. Studies with squalene synthase inhibitors have also revealed a number of previously unrecognized pathways that control the expression of many enzymes capable of degrading the majority of the synthetic intermediates produced during cholesterol synthesis prior to ring closure (i.e. squalene-2,3-oxide cyclization to generate lanosterol) (Fig. S1). Following ring closure, during the synthesis of steroid hormones (e.g. estrogen, testosterone, cortisol), enzymes expressed in steroidogenic cells can remove carbons from the C-17 side chain, and add, modify, or remove substituents at C-3,-4,-5,-7,-11,-17,-18^38^. In the liver, hepatic cytochrome P450s (e.g. CYP3A4, CYP7A1, CYP8B1, CYP27A1) modify cholesterol in the generation of bile acids (Fig. S1). We concluded that humans cannot degrade cholesterol because humans do not have the enzymes needed to open the cholestane ring.

**Figure 1:**
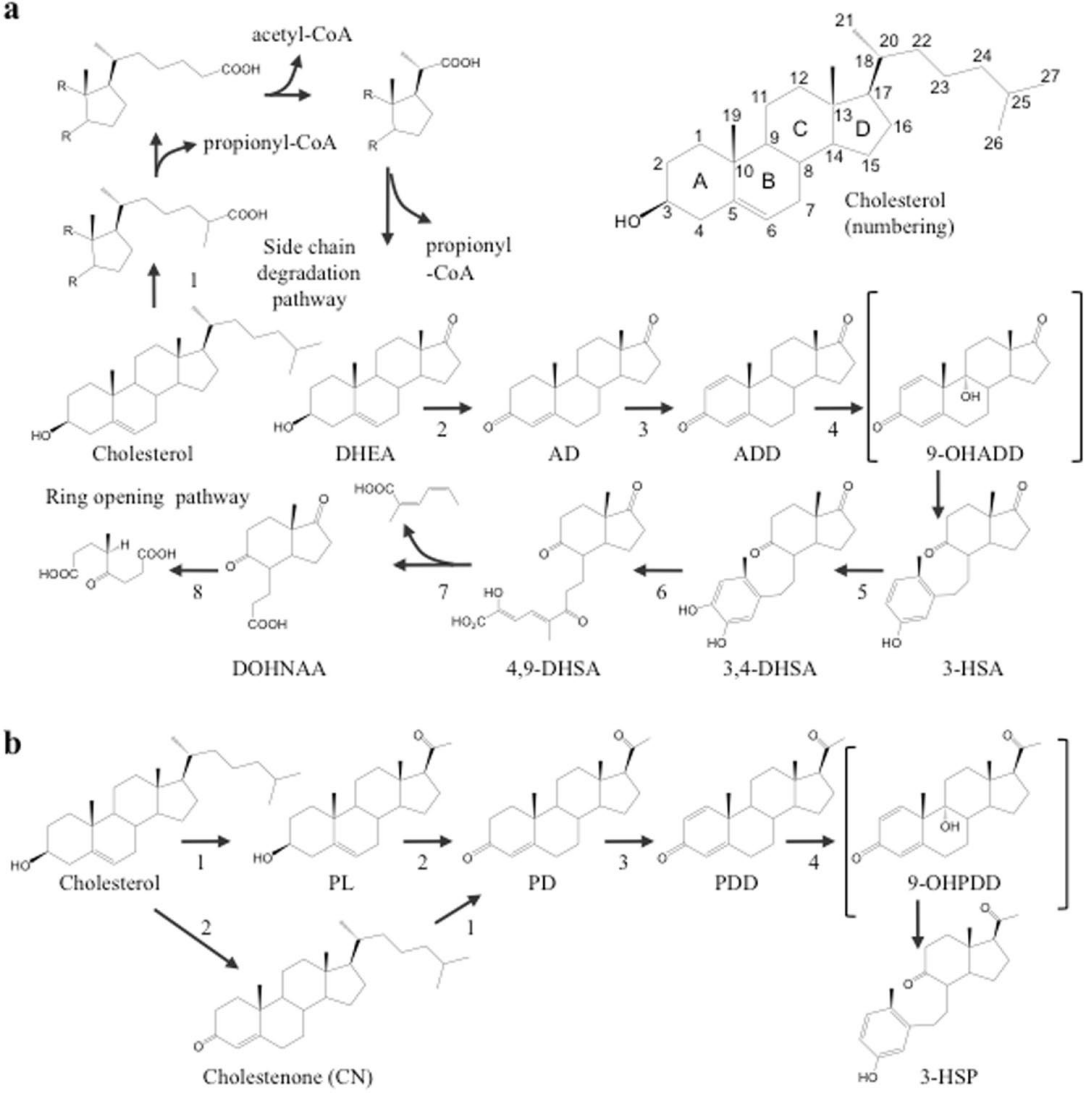
*Cholesterol catabolism pathways*. (**a**) Cholesterol catabolism pathway in *Actinomycetes*. *Mycobacterium tuberculosis* and some species of *Rhodococcus* have the capacity to catabolize cholesterol as a source of carbon and energy. In these strains, two catabolic pathways have been identified. One (step 1) is the side chain degradation pathway, in which the aliphatic side chain on C-17 is removed in a process similar to beta-oxidation. The second is the four-ring degradation pathway (steps 2–8). In ring degradation, three enzymes, cholesterol-3-OH-dehydrogenase (step 2), 3-ketosteroid Δ^1^-dehydrogenase (step 3), and 3-ketosteroid-9α-hydroxylase (step 4) catalyze B-ring opening and A-ring aromatization to produce 3-hydroxy-9,10-seconandrost-1,3,5(10)-triene-9,17-dione (3-HSA). Three additional enzymes (HasAB, step 5; HasC, step 6; HasD; step 7) then generate 9,17-dioxo-1,2,3,4,10,19-hexanorandrostan-5-oic acid (DOHNAA) via oxygenolytic cleavage of ring A. (**b**) Theoretical pathway for cholesterol catabolism in humans. Steroidogenic cells express cytochrome p450s that can remove the C-17 side chain during steroid hormone biosynthesis (step 1). However, humans lack enzymes needed to degrade the cyclopentanoperhydrophenanthrene (cholestane) ring. To allow B ring opening in human cells we humanized cholesterol-3-OH-dehydrogenase (CholD; to catalyze step 2), 3-ketosteroid Δ^1^-dehydrogenase (Δ^1^-KstD; to catalyze step 3) and 3-ketosteroid-9α-hydroxylase (Kst-9αH; to catalyze step 4). Brackets designate unstable intermediate compounds that degrade spontaneously. Abbreviations: DHEA, 3β-hydroxyandrost-5-ene-17-one; AD, androst-4-ene-3,17-dione; ADD, androsta-1,4-diene-3,17-dione; 3,4-DHSA, 3,4-dihydroxy-9,10-seconandrost-1,3,5(10)-triene-9,17-dione; 4,9-DSHA, 4,5–9,10-diseco-3-hydroxy-5,9,17-trioxoandrosta-1(10),2-diene-4-oic acid; DOHNAA, 9,17-dioxo-1,2,3,4,10,19-hexanorandrostan-5-oic acid; PL, 3β-hydroxypregn-5-en-20-one; PD, pregn-4-ene-3,20-dione; PDD, pregn-1,4-diene-3,20-dione; 3-HSP, 3-hydroxy-9,10-secopregn-1,3,5(10)-triene-9,20-dione; 9-OHPDD, 9-hydroxypregn-1,4-diene-3,20-dione. The pathways in part A are based, in part, on the studies of Van der Geize *et al*.^53^.

Like humans, most bacteria cannot catabolize cholesterol. However, during chronic infection *Mycobacterium tuberculosis* can catabolize cholesterol to generate carbon and energy while contained in phagosomes of macrophages^39,40^. Further study revealed that in addition to *M*. *tuberculosis*, certain *Rhodococcus sp*. and *Sterolibacterium sp*. are also capable of degrading cholesterol and cholesterol derived sterols^41,42^. Notably, both *Mycobacteria* and *Rhodococci* have similar enzymes that catalyze cholestane B-ring opening (Fig. 1). These observations lead us to the provocative hypothesis that if we could enable controlled cholesterol catabolism in human cells, surplus cholesterol could be degraded. In theory, if cholesterol catabolism is introduced in the liver, low levels of hepatic cholesterol should induce LDL receptor expression, in turn, lowering the level of circulating LDLs. If introduced into monocytes, after migrating to inflamed atherosclerotic lesions and differentiating into macrophages, the cholesterol-catabolizing macrophages may be able to prevent or even reverse the atherosclerotic process. To test this hypothesis, here we describe the generation of three synthetic humanized enzymes predicted to enable cholestane B-ring opening [cholesterol-3-OH-dehydrogenase (CholD), 3-ketosteroid Δ^1^-dehydrogenase (Δ^1^-KstD), and 3-ketosteroid-9α-hydroxylase (Kst-9αH)]. After documenting the desired catalytic activity of the recombinant enzymes *in vitro*, we then further characterize one (Δ^1^-KstD_R_; designed based on *Rhodococcus erythropolis* KstD1^43–45^) following stable expression in two different human cell lines (Hep3B and U-937). Our studies reveal that when expressed in human cells Δ^1^-KstD_R_ introduces a double bond between the C-1 and C-2 atoms of 3-ketosteroids. This represents an integral step needed for cholestane A-ring aromatization and B-ring opening for which humans have no known ortholog.

## Results

### Synthetic, humanized Cholesterol-3-OH-Dehydrogenase (CholD), 3-Ketosteroid Δ^1^-Dehydrogenase (Δ^1^-KstD), and 3-Ketosteroid-9α-Hydroxylase (Kst-9αH) have the expected catalytic activities when expressed as recombinant enzymes

Actinomycetes have two pathways associated with cholesterol catabolism (i.e. the side chain degradation pathway and the B-ring opening pathway; Fig. 1a). Humans have enzymes that can remove the C-17 side chain. Therefore, our hypothesis was that if we introduced the three missing enzymes (CholD, Δ^1^-KstD, and Kst-9αH) we could achieve B-ring opening in human cells (Fig. 1b). To test this hypothesis, we first designed and synthesized expression constructs encoding humanized orthologs of all three enzymes (Tables S1–S4). We next tested the activity of each recombinant enzyme following expression in *E*. *coli*. For these studies, reverse-phase high-performance liquid chromatography (RP-HPLC) and liquid chromatography-tandem mass spectrometry (LC-MS/MS) based methods were used to measure the ability of Δ^1^-KstD to introduce a double bond between C-1 and C-2 (Figs S2–S4). Similar methods were used to assess CholD activity (i.e. the generation of a C-3 ketone, which is needed for Δ^1^-KstD activity; Figs S5, S6), and Kst-9αH activity (i.e. the addition of a hydroxyl to C-9 in ring B; Figs S7, S8). When a hydroxyl is added at C-9 to Δ^1^-Δ^4^-3-ketosteroids, the B ring becomes unstable and spontaneously opens (Fig. 1b). In addition, the side chain degradation pathway in bacteria generates catabolites with a ketone at C-17 (e.g. androst-4-ene-3,17-dione; AD), whereas CholD generates pregn-4-ene-3,20-dione (PD) in our theoretical pathway. Because PD has two additional carbons and a ketone at C-20 (Fig. 1b), we also needed to determine if our synthetic enzymes would act upon substrates with these structural differences.

Like most bacteria, *E*. *coli* lack both anabolic and catabolic activity against most tetracyclic sterols. This allowed us to rapidly test the catalytic activity and the substrate specificity of all three synthetic enzymes using clarified lysates generated from *E*. *coli* transformed with expression plasmids encoding each of the enzymes. Catabolites were identified using [^14^C or ^13^C]-spiked substrates and RP-HPLC, employing established methods^46^. We started by synthesizing an expression plasmid capable of generating a humanized CholD (Table S1). CholD was designed based on the amino acid sequence of the CholD gene from *Mycobacterium tuberculosis* (Table S1). Recombinant CholD expressed in *E*. *coli* was active against both cholesterol, generating 5-cholesten-3-one (cholestenone; CN; Fig. S5), and 3β-hydroxypregn-5-en-20-one (PL), generating PD. This indicted that the hydrophobic C-17 side chain was not necessary for substrate recognition by CholD (Fig. S6).

**Figure 2:**
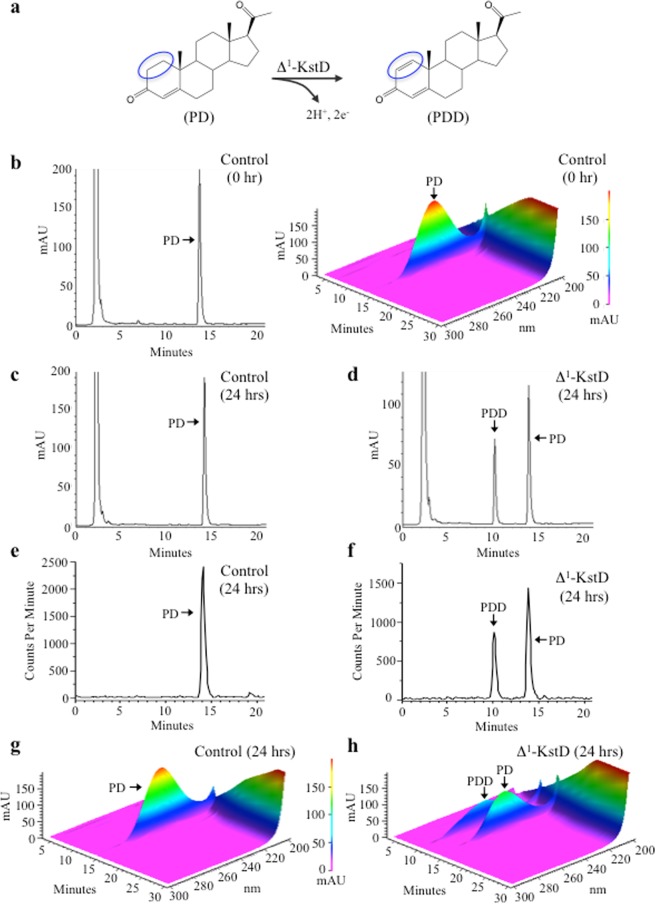
Humanized 3-ketosteroid Δ^1^-dehydrogenase (Δ^1^-KstD) catalyzes the C-1 and C-2 dehydrogenation of pregn-4-ene-3,20-dione (PD) generating pregn-1,4-diene-3,20-dione (PDD). Clarified lysates produced in an identical manner from either *E*. *coli* expressing Δ^1^-KstD or *E*. *coli* transformed with an empty (pUC19) expression plasmid (control) were incubated with C4-[^14^C] labeled PD. Lipids were extracted at 0 and 24 hours and analyzed by RP-HPLC as described in the methods. (**a**) Chemical structures and reaction summary. (**b**) Representative HPLC chromatogram of the 0-hour time point showing only PD (λ_max_: 245 nm; *t*_*r*_ = 13.8 min) in control extracts with the 3-D chromatogram showing the spectral data (λ_300-200 nm_) plotted against time and absorption (mAU) shown on the right. (**c**) Representative HPLC chromatogram of the 24-hour time point showing only PD (λ_max_: 245 nm; *t*_*r*_ = 13.8 min) in control extracts (**d**) Representative HPLC chromatogram of the 24 hour time point showing PD (λ_max_: 245 nm; *t*_*r*_ = 13.8 min) and PDD (λ_max_: 247 nm; *t*_*r*_ = 10.0 min) in extracts from *E*. *coli* expressing Δ^1^-KstD treated in an identical manner as controls. (e) [^14^C] measured by the in-line scintillation detector corresponding to the chromatograms shown in (**c**). (**f**) [^14^C] measured by the in-line scintillation detector corresponding to the sample run shown in (**d**). (**g**) 3-D chromatogram showing the spectral data (λ_300-200 nm_) plotted against time and absorption (mAU) of the sample run shown in (**c**). (**h**) 3-D chromatogram showing the spectral data (λ_300-200 nm_) plotted against time and absorption (mAU) of the sample run shown in (**d**). Chromatograms of aliquots taken at timed intervals are shown in supplemental data (Fig. S3).

We next tested two synthetic Δ^1^-KstDs, one developed based on the KstD1 gene of *Rhodococcus erythropolis* (Δ^1^-KstD_R_; Table S2), and the other based on the Δ^1^-KstD encoded by the acmB gene of *Sterolibacterium denitrificans* (Δ^1^-KstD_A_; Table S3). For initial studies, we used a radiolabeled test substrate (C4-[^14^C] pregn-4-ene-3,20-dione (PD; 100 μM - 20 nCi C4-[^14^C]), which when converted to pregn-1,4-diene-3,20-dione (PDD) simultaneously tested the Δ^1^-dehydrogenase activity of each enzyme as well as the ability of Δ^1^-KstD to utilize sterols with a C-20 ketone (Figs 2 and S3). As expected, when lysates were generated from *E*. *coli* transformed with an empty control vector (pUC19), RP-HPLC analysis revealed that PD (λ_max_: 245 nm; *t*_*r*_ = 13.8 min) remained stable for >24 hours (Fig. 2a–c,e,g). In contrast, when lysates generated from *E*. *coli* expressing Δ^1^-KstD_R_ were incubated with PD under identical conditions, there was a time dependent decrease in the amount of PD with a concomitant increase in [^14^C] pregn-1,4-diene-3,20-dione (PDD; λ_max_: 247 nm; *t*_*r*_ = 10.0 min) (Figs 2d,f,h, S3). The generation of PDD was confirmed by MS1/MS2 mass spectrometry (Fig. S4). Based on the [^14^C]-RP-HPLC data, no additional metabolites were observed in either lysate (Fig. 2e,f). Thus, the bacterial lysate also proved to be an efficient means to generate [^14^C]-labeled PDD, which was needed to test the ability of the humanized Kst-9αH to utilize sterol substrates containing a C-20 ketone.

The third missing enzyme, 3-ketosteroid-9α-hydroxylase (Kst-9αH) was designed based on the genes designated as KshA/B in *Rhodococcus rhodochrous* (Table S4). Consistent with studies conducted with enzymes from *Actinomycetes*, Kst-9αH demonstrated only modest activity against cholestenone (CN) (Fig. S7) and more robust activity against PD (Fig. S8). Δ^1^-KstD_A_ was also active against CN (Fig. S9) whereas Δ^1^-KstD_R_ preferred PD (Fig. 2). This trend was also observed when the enzymes were combined *in vitro* [i.e. when extracts for *E*. *coli* expressing either Δ^1^-KstD_R_ or Δ^1^-KstD_A_ were combined with Kst-9αH, PD was completely converted to the end product 3-HSP (Fig. S10)]. In contrast, although 3-HSC was clearly generated by the mixture of CholD/Δ^1^-KstD_A_/Kst-9αH, substantial [^14^C]-labeled residual cholesterol, CN, and other intermediate catabolites were still present in the extracts after 24 hours (Fig. S11). Again, as expected in control *E*. *coli* lysates, cholesterol (Fig. S12), CN (Fig. S13), and PD (Fig. S14) are all extremely stable, and no evidence of metabolism was detected after 24 hours. These studies revealed that the synthetic humanized enzymes have the desired catalytic activity, and that their combined activity was sufficient to open the B ring of cholesterol. However, in human cells cholesterol homeostasis is highly regulated. Synthesis is controlled by the regulation of HMG-CoA reductase at many levels, and uptake can be mediated by many transport mechanisms (e.g. LDL-Rs, SR-AI/AII, CD36, CD68, LOX1). Therefore, next we determined if the synthetic enzymes could be made functional when expressed in human cells.

Although transient transfection studies suggested CholD was active when expressed in human cells, our attempts to generate stable human cell lines that expressed CholD alone failed. In addition, when cholestenone was added to control cells in culture we observed toxicity, with an LD_50_ ~75 μM after 72 hours (Fig. S15). These studies do not demonstrate that the CholD-transformed cells died due to the over production of cholestenone generated by the introduction of CholD activity. However, we easily generated human cell lines that expressed Δ^1^-KstD_R_, Kst-9αH, or both Δ^1^-KstD_R_, Kst-9αH, which have no endogenous substrates without the upstream activity of CholD. Therefore, we predict that the downstream enzymes need to have equal or greater activity when compared to the enzymes acting upstream to allow the pathway to “flow” and to prevent the accumulation of toxic intermediates. To obtain estimates of substrate binding affinity and turnover rates, we next conducted kinetic studies starting with Δ^1^-KstD_R_.

### Δ^1^-KstD_R_ kinetic studies

For initial kinetic analysis, we expressed Δ^1^-KstD_R_ as an N-terminal FLAG His-patch thioredoxin fusion protein (HP-THX-FLAG-Δ^1^-KstD_R_) and partially purified Δ^1^-KstD_R_ using immobilized metal affinity chromatography (IMAC) (Fig. S16). Active fractions (19–22) were identified using an in-gel nitrotetrazolium blue assay (NTB; Fig. S16a, S17). SDS-PAGE, Coomassie staining, and western analysis identified a protein with the predicted (100 kDa) size of HP-THX-FLAG-Δ^1^-KstD_R_ in the fractions with NTB activity (Fig. S15b,d). Δ^1^-KstD_R_ eluted in 25 mM Tris-HCl, pH 7.5, containing 500 mM NaCl and 120 mM imidazole, and elution fraction 21 contained a reasonable yield (0.385 mg/mL) of ~80% pure Δ^1^-KstD_R_ (Fig. S15c).

To verify Δ^1^-KstD_R_ was indeed responsible for the dehydrogenase activity generated in the NTB assay, 0.77 μg IMAC purified protein was incubated with 10 μM PD at 37 °C. After four hours, the reaction mixtures were extracted with ethyl acetate and analyzed by RP-HPLC, revealing a ~90% reduction of substrate (PD; λ_max_: 245 nm, *t*_*r*_ = 13.8 min) associated with the concomitant production of PDD (λ_max_: 247 nm, *t*_*r*_ = 10.0 min) (Fig. S16e–h). These data demonstrate that the synthetic Δ^1^-KstD_R_ is indeed active against PD.

**Figure 3:**
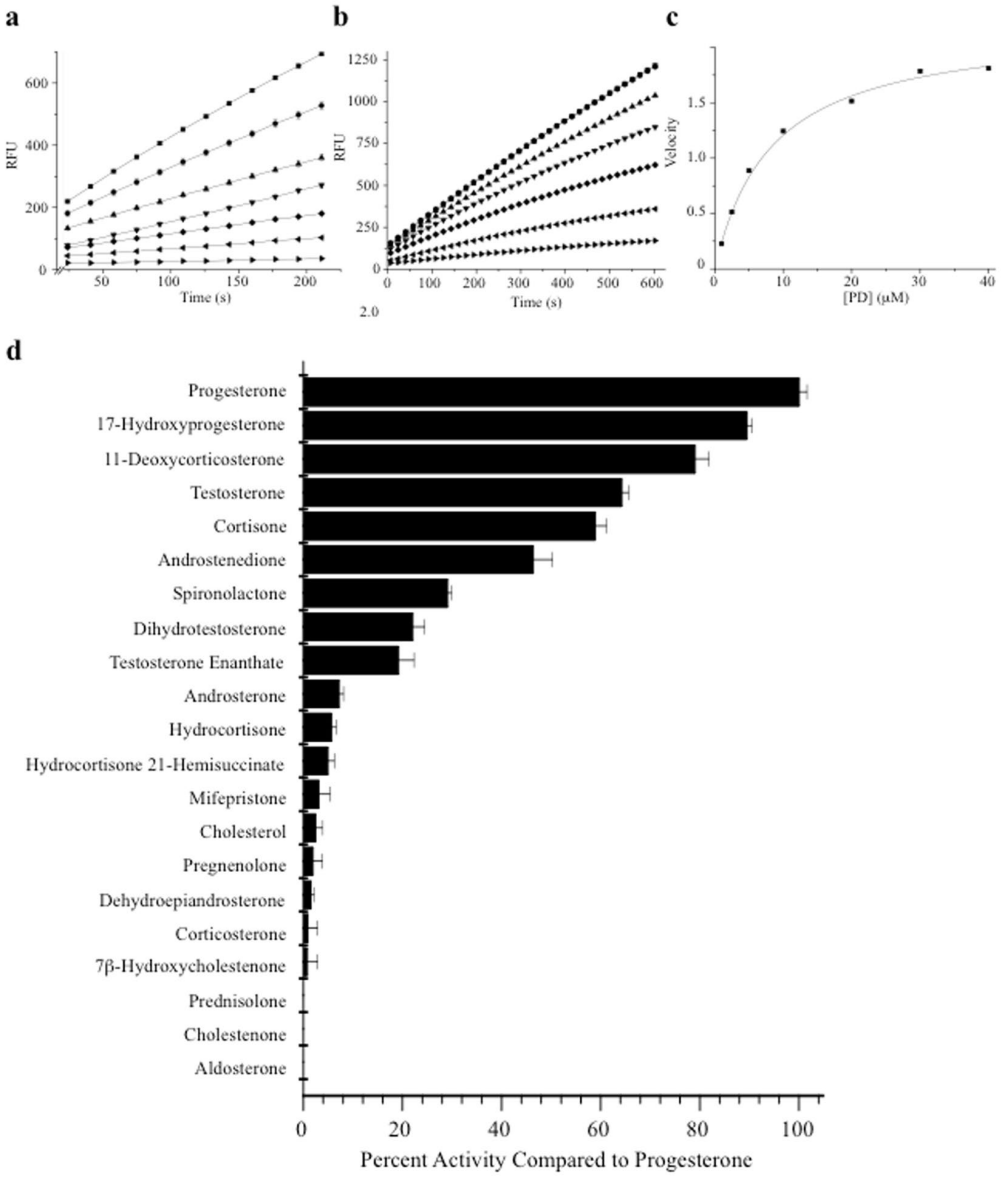
Δ^1^-KstD_R_ kinetic analysis and substrate specificity screen. (**a**) Δ^1^-KstD titration curves illustrating how changes in enzyme concentration (2.1 nM; square, 1.6 nM; circle, 1.1 nM; upward facing triangle, 0.55 nM; downward facing triangle, 0.37 nM; diamond, 0.19 nM; left facing triangle, and 0.05 nM; right facing triangle) affect the fluorescence (RFU) measured from the reaction product with respect to time using 20 μM of substrate (PD). Resorufin fluorescence (Fig S18) was measured at 17 sec intervals for 3.5 min. Each data point represents the mean of three replicates at the indicated time and concentration (mean ± SE; n = 3). (**b**) Reaction progress curves generated with fixed concentrations of Δ^1^-KstD (0.55 nM) and varying concentration of substrate (PD): (40 μM; square, 30 μM; circle, 20 μM; upward facing triangle, 10 μM; downward facing triangle, 5 μM; diamond, 2.5 μM; left facing triangle, and 1 μM; right facing triangle). Resorufin fluorescence was measured at 17 sec intervals for 10 minutes (mean ± SE, n = 8). (**c**) Kinetic analysis of PD C-1 and C-2 A-ring desaturation by Δ^1^-KstD. Initial velocities of the reactions were determined from the linear portion of the 0.55 nM Δ^1^-KstD progress curves shown in panel (**b**) by least squares analysis and plotted against the substrate concentration. K_M_ (8.3 +/−0.5 μM) was determined by non-linear curve fitting of the data to the Michaelis-Menten equation. All reactions contained (20 μM) resazurin. All reactions were conducted in a 96-well format (V_f_ = 300 μL) using a BioTek Synergy 2 plate reader (excitation 540 ± 25 nm; emission 620 ± 40 nm). (**d**) Δ^1^-KstD_R_ substrate specificity screen. The substrate preference of Δ^1^-KstD was assessed by testing steroids using the resazurin based fluorescence assay. Reactions containing 5.35 nM Δ^1^-KstD, 20 μM resazurin, and 20 μM of the indicated substrates were conducted as described in methods. The data shown represents the mean activity of four replicates normalized to the activity demonstrated against PD (mean ± SE, n = 4). In this experiment, all compounds were tested head-to-head with the same preparation of Δ^1^-KstD in a 96 well format.

For further kinetic analysis, a direct-coupled flurometric assay was developed based on resazurin, which is a weakly fluorescent redox dye that is irreversibly reduced when it accepts protons released from a donor molecule (Fig. S18). Reduction of resazurin results in the formation of the highly fluorescent product, resorufin. In this assay, Δ^1^-KstD_R_ removes and donates two protons and two electrons from C-1 & C-2 of ring-A directly to resazurin, resulting in the formation of resorufin and water (Fig. S18). This reduction can be measured by monitoring the increase in fluorescence intensity with time, allowing the assessment of the initial rates of Δ^1^-KstD_R_ substrate conversion. The linear range of the assay was determined for several concentrations of Δ^1^-KstD_R_ (0.05, 0.19, 0.37, 0.55, 1.1, 1.6, 2.12 nM) revealing good linearity for 0.55 nM Δ^1^-KstD_R_ for >3.5 minutes (Fig. 3a). Next, enzyme progress curves were generated by incubating 0.55 nM Δ^1^-KstD_R_ with increasing concentrations of PD (1, 2.5, 5, 10, 20, 30, 40 μM) (Fig. 3b). Nonlinear regression analysis of the data (Fig. 3c) revealed the partially purified enzyme has a K_M_ of 8.3 +/−0.5 μM with PD as the substrate.

The resazurin assay was next used to test the ability of Δ^1^-KstD_R_ to act on cholesterol or 20 cholesterol-derived steroids, including a number of pregnane-, androstane-, and cholestane-based compounds (Fig. 3d). In addition to PD, Δ^1^-KstD_R_ demonstrated activity against nine 3-keto containing substrates (ranked highest to lowest in activity: progesterone, 17-hydroxyprogesterone, 11-deoxycorticosterone, testosterone, cortisone, androstenedione, spironolactone, dihydrotestosterone, and testosterone enanthate). Little or no activity was observed against 3-hydroxy steroids, indicating Δ^1^-KstD_R_ requires the 3-ketone on ring-A. Δ^1^-KstD_R_ also demonstrated little activity against substrates containing a long alkyl C-17 side chain, including cholestenone, which contains the desired C-3-ketone.

### Δ^1^-KstD_R_ expression in Hep3B cells

**Figure 4:**
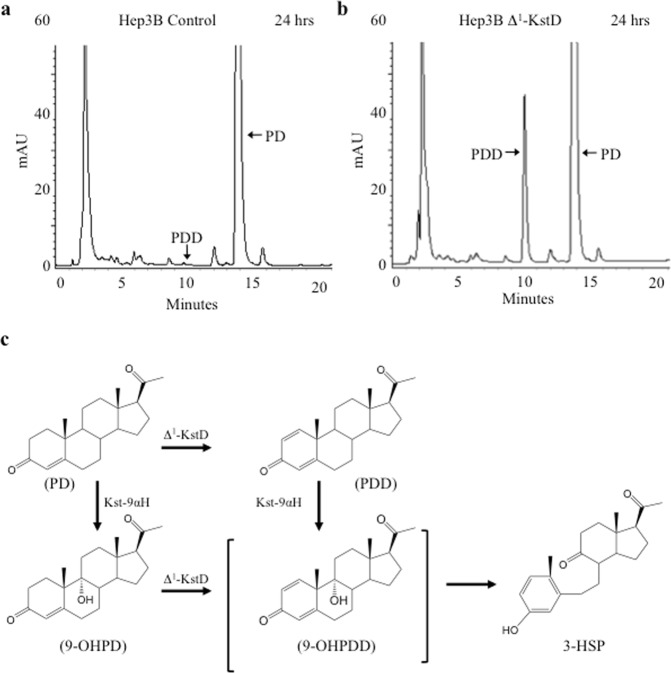
Δ^1^-KstD_R_ expression in Hep3B cells enables novel catabolic activity. Representative HPLC chromatograms (λ 245 nm) showing PD (*t*_*r*_ = 13.8 min) in control Hep3B cells (**a**) and Hep3B expressing Δ^1^-KstD (**b**) after 24 showing the accumulation of PDD (λ_max_: 247 nm; *t*_*r*_ = 10.0 min) only in the Hep3B cells expressing Δ^1^-KstD. Additional data and images showing [^14^C] trace detected by the in-line scintillation detector corresponding to the chromatograms are provided in Figs S20 and S21. For all experiments, Hep3B controls (left) or Hep3B Δ^1^-KstD cells (right) were incubated with 15.7 μg (10 μM) PD spiked with 100 nCi C4-[^14^C] labeled PD (*t*_*r*_ = 13.8 min). (**c**) Structures of substrates, products, and a diagram of the reaction summary following catabolism of PD generating PDD and catabolism of 9-hydroxypregn-4-ene-3,20-dione (9-OHPD) generating 3-hydroxy-9,10-secopregn-1,3,5(10)-triene-9,20-dione (3-HSP).

Having established that the recombinant Δ^1^-KstD_R_ has the desired catalytic activity when expressed in *E*. *coli*, (i.e. K_M_ below the predicted cytotoxic concentration of cholestenone) we now needed to determine whether the synthetic enzyme could correctly fold and demonstrate sufficient catalytic activity when expressed in human cells. To test this, we subcloned Δ^1^-KstD_R_ into lentiviral expression vectors, which were used to generate stable Δ^1^-KstD_R_ expressing cell lines with expression driven by either PGK- or CMV-derived promoters. Based on stable expression levels, a CMV-driven Δ^1^-KstD_R_-Hep3B cell line was chosen for further analysis (Fig. S19). For these studies, equal numbers of control (non-transduced Hep3B cells) and Δ^1^-KstD_R_-Hep3B cells were plated in replicate dishes, grown until confluent and treated with 10 μM PD spiked with 100 nCi C4-[^14^C] PD. After 24, 48, and 72 hours of incubation, the cells and media were extracted with ethyl acetate, and the lipids were analyzed by RP-HPLC. The spectral data revealed pregn-1,4-diene-3,20-dione (PDD; t_r_ = 10.0 minutes; λ_max_: 247 nm) accumulation in a time dependent manner over the 72-hour time course in the Δ^1^-KstD_R_-Hep3B cells (e.g. Figs 4; S20). In contrast, control Hep3B cells lacked activity needed to produce PDD, as illustrated by the absence of a 10-minute peak (Fig. 4a). PDD production was confirmed by MS1/MS2 mass spectrometry analysis (Fig. S21).

### 3-hydroxy-9,10-secopregn-1,3,5(10)-triene-9,17-dione (3-HSP) formation in Hep3B cells expressing Δ^1^-KstD_R_

**Figure 5:**
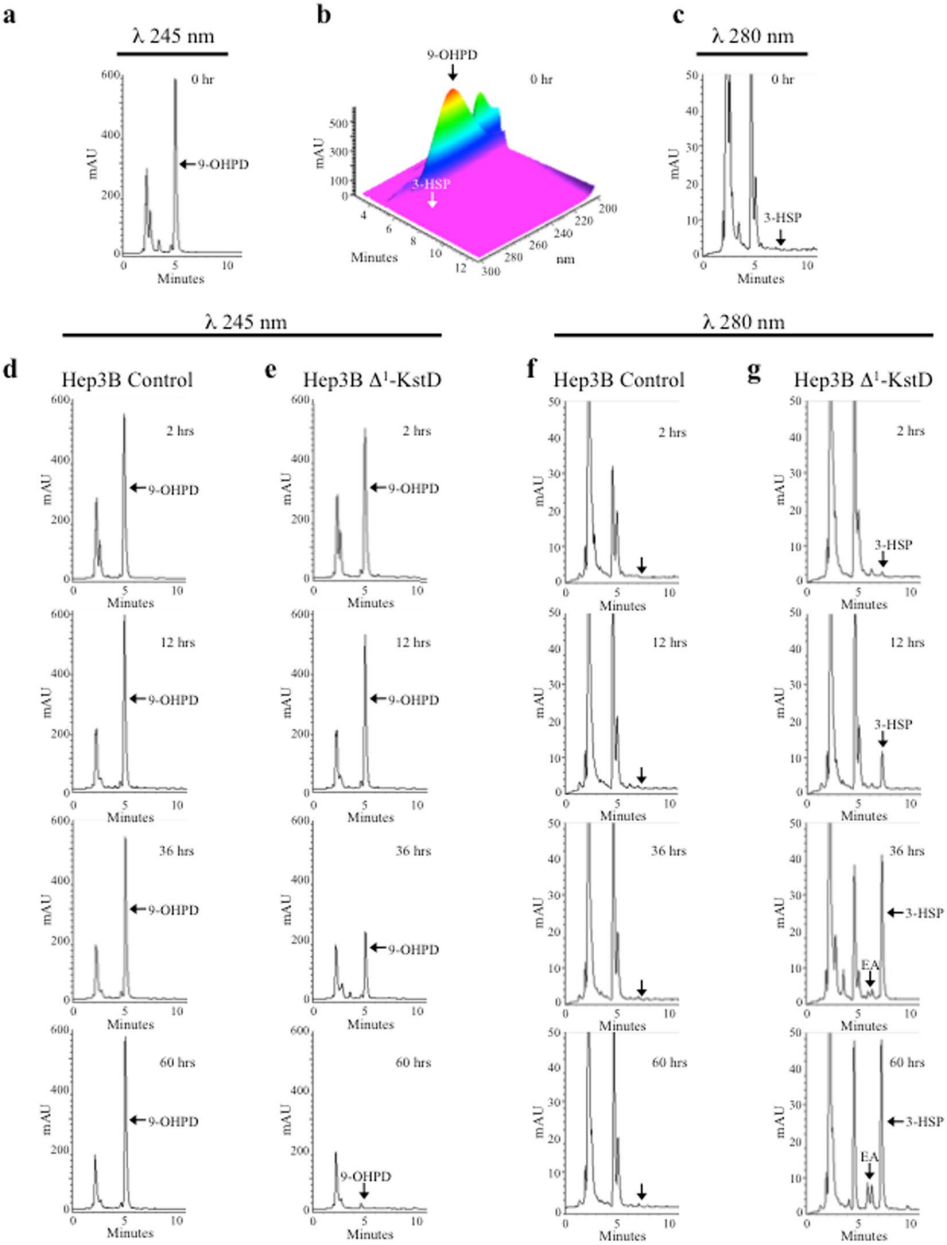
Cholestane ring opening in human cells. In Hep3B Δ^1^-KstD cells, catabolism of PD forms PDD and catabolism of 9-hydroxypregn-4-ene-3,20-dione (9-OHPD) forms 3-hydroxy-9,10-secopregn-1,3,5(10)-triene-9,20-dione (3-HSP). (**a**) Representative HPLC chromatogram showing 9-OHPD (λ_max_: 245 nm; *t*_*r*_ = 5.2 min) generated from control cell lysates. (**b**) 3-D chromatogram showing the spectral data (λ_300-200 nm_) plotted against time and absorption (mAU) of the sample run shown in (**a**). (**c**) Representative HPLC chromatogram showing the lack of 3-HSP (λ_max_: 280 nm; *t*_*r*_ = *7*.2 min) at 0 hours in control cell lysates. (**d**) Representative HPLC chromatograms showing the stability of 9-OHPD in control cells. (**e**) Representative HPLC chromatograms documenting the time dependent loss of 9-OHPD (λ_max_: 245 nm; *t*_*r*_ = 5.2 min) with time in Hep3B Δ^1^-KstD cells. (**f**) Representative HPLC chromatograms showing the lack of 3-HSP formation with time in control cells. (**g**) Representative HPLC chromatograms documenting the time dependent increase of 3-HSP (λ_max_: 280 nm; *t*_*r*_ = 7.2 min) in Hep3B Δ^1^-KstD cells.

**Figure 6:**
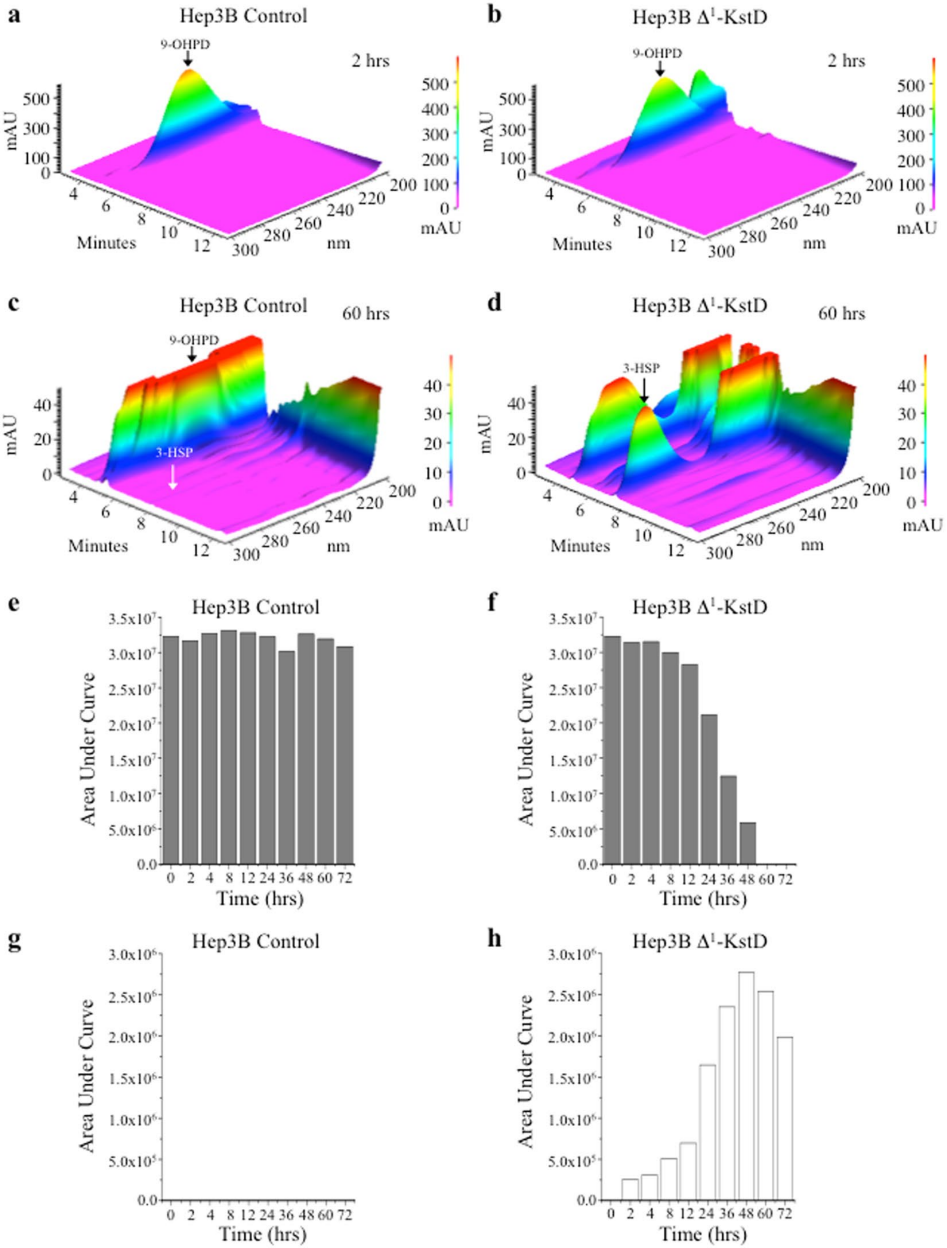
Summary of cholestane ring opening in Hep3B Δ^1^-KstD cells. (**a**–**d**) 3-D chromatogram showing the spectral data (λ_300-200 nm_) plotted against time and absorption (mAU) of the sample run shown in (Fig. 5d,f) and (Fig. 5e,g) at 2 hours (**a**,**b**) and 60 hours (**c**,**d**), respectively. (**e**–**h**) Summary of the time-course studies plotted as bar graphs showing the stability of 9-OHPD (**e**) and the lack of 3-HSP (**g**) accumulation in Hep3B control cells. (**f** and **h**) Bar graphs showing the time dependent loss of 9-OHPD (**f**) and the concomitant accumulation of 3-HSP (**h**) in Hep3B Δ^1^-KstD cells are shown. Each bar represents the area under the curve obtained from the RP-HPLC-chromatogram of compounds separated using a C-18 column and identified based on established retention times and corresponding spectral data, as described in Methods. In the Hep3B Δ^1^-KstD cell line, 3-HSP accumulates until the supply of 9-OHPD is depleted (**f**,**h**). Then 3-HSP diminishes with time, suggesting endogenous enzymes may degrade 3-HSP.

From studies conducted with the *M*. *tuberculosis* ortholog of KstD, it was predicted that Δ^1^-KstD will utilize either PD or another catabolite, 9-hydroxypregn-4-ene-3,20-dione (9-OHPD; λ_max_ 245 nm; t_r_ = 5.2 min) as a substrate. 9-OHPD is generated by Kst-9αH, which can also use PD as a substrate (Fig. S22). The desaturation of C-1 and C-2 in 9-OHPD leads to the generation of an unstable product, 9-hydroxypregn-1,4-diene-3,20-dione (9-OHPDD), which can also be generated by the actions of Kst-9αH on PDD (Fig. 4c). The B-ring of 9-OHPDD undergoes spontaneous non-enzymatic cleavage with concomitant aromatization of ring-A to form the product 3-hydroxy-9,10-secopregn-1,3,5(10)-triene-9,17-dione (3-HSP). As a consequence of ring-A aromatization, 3-HSP demonstrates a characteristic spectral absorbance shift (λ_max_ 245 nm to λ_max_ 280 nm) providing an easily detectable indicator that B-ring opening has been achieved (Fig. S10). As illustrated in Fig. 5a–g and summarized in Fig. 6e–h, Hep3B cells expressing Δ^1^-KstD_R_ displayed a time dependent decrease in 9-OHPD (λ_max_ 245 nm; t_r_ = 5.2 min; compare Fig. 5d and e) associated with a concomitant increase in a new peak with a retention time of 7.2 minutes and a λ_max_ of 280 nm, characteristic of 3-HSP (compare Fig. 5f and g). In contrast to PD, quantitative analysis of the 9-OHPD in control cells revealed that 9-OHPD levels remained stable for at least 72 hours (Figs 5d and 6e). When Δ^1^-KstD_R_-Hep3B cells were provided with a single 10 μM dose of 9-OHPD, nearly all of the 9-OHPD substrate had been catabolized by 60 hours (Figs 5e and 6f). No 3-HSP was detected in control cells (Figs 5f and 6g) and maximal accumulation of 3-HSP was observed at 48 hours in the Δ^1^-KstD_R_-Hep3B cell extracts (Figs 5g and 6h). The reduction in 3-HSP levels (48–72 hr) indicates Hep3B cells have endogenous metabolic capability to further act upon the ring opened catabolites (Figs 5g and 6h). This endogenous activity has not yet been characterized further. Together, these data indicate that, when expressed in Hep3B cells, Δ^1^-KstD_R_ can utilize either PD or 9-OHPD as substrates.

### PDD (pregn-1,4-diene-3,20-dione) and 3-HSP (3-hydroxy-9,10-secopregn-1,3,5(10)-triene-9,17-dione) generation in Δ^1^-KstD_R_-expressing U937-cells

**Figure 7:**
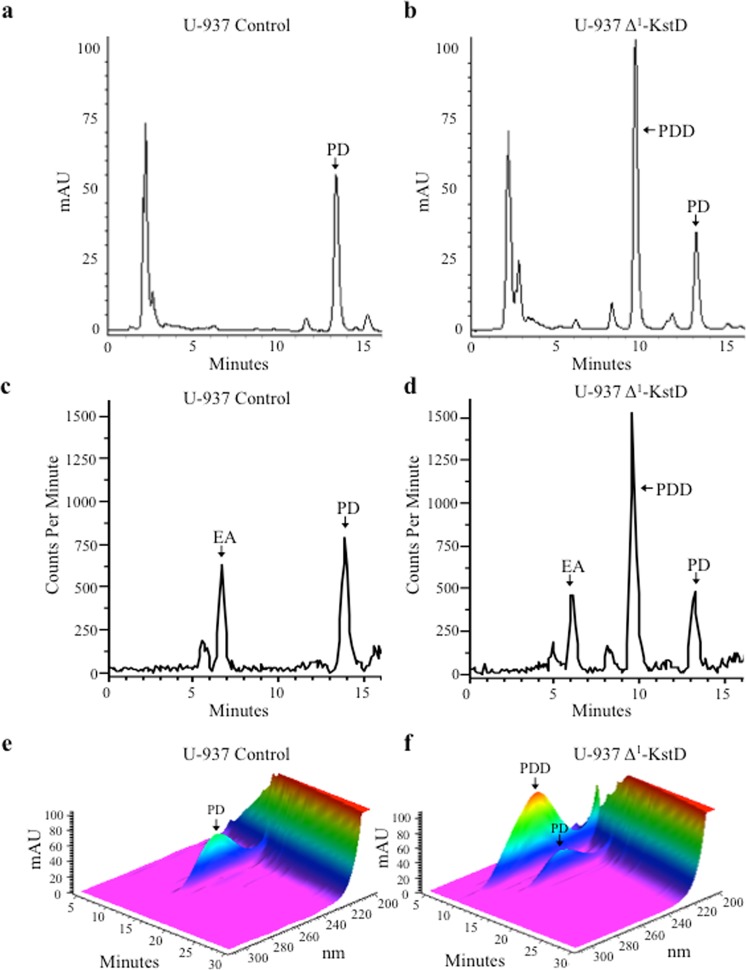
U-937-derived macrophages expressing Δ^1^-KstD catalyze the C-1 and C-2 dehydrogenation of pregn-4-ene-3,20-dione (PD) generating pregn-1,4-diene-3,20-dione (PDD). Representative HPLC chromatograms (λ_245 nm_) showing PD (*t*_*r*_ = 13.8 min) in controls (**a**,**c**,**e**) and U-937-derived macrophages expressing Δ^1^-KstD (**b**,**d**,**f**) after 72 hours showing the accumulation of PDD (λ_max_: 247 nm; *t*_*r*_ = 10.0 min) only in the U-937 cells expressing Δ^1^-KstD. (**c**) Representative image showing [^14^C] trace detected by the in-line scintillation detector corresponding to the chromatogram shown in panel (**a**) revealing no PDD formation but a novel peak representing endogenous activity (EA; *t*_*r*_ = 6.5 min) in control cells. (**e**) 3-D chromatogram showing the spectral data (λ_300-200 nm_) plotted against time and absorption (mAU) of the sample run shown in (**a**). (**d**) [^14^C] trace of the chromatogram shown in panel (**b**), showing PDD (*t*_*r*_ = 10.0 min) and similar endogenous activity (EA; *t*_*r*_ = 6.5 min). (**f**) 3-D chromatogram showing the spectral data (λ_300-200 nm_) plotted against time and absorption (mAU) of the sample run shown in (**b**). For all experiments, U-937 control (left) or U-937 Δ^1^-KstD cells (right) were incubated with 15.7 μg (10 μM) PD spiked with 100 nCi C4-[^14^C] labeled PD (*t*_*r*_ = 13.8 min).

**Figure 8:**
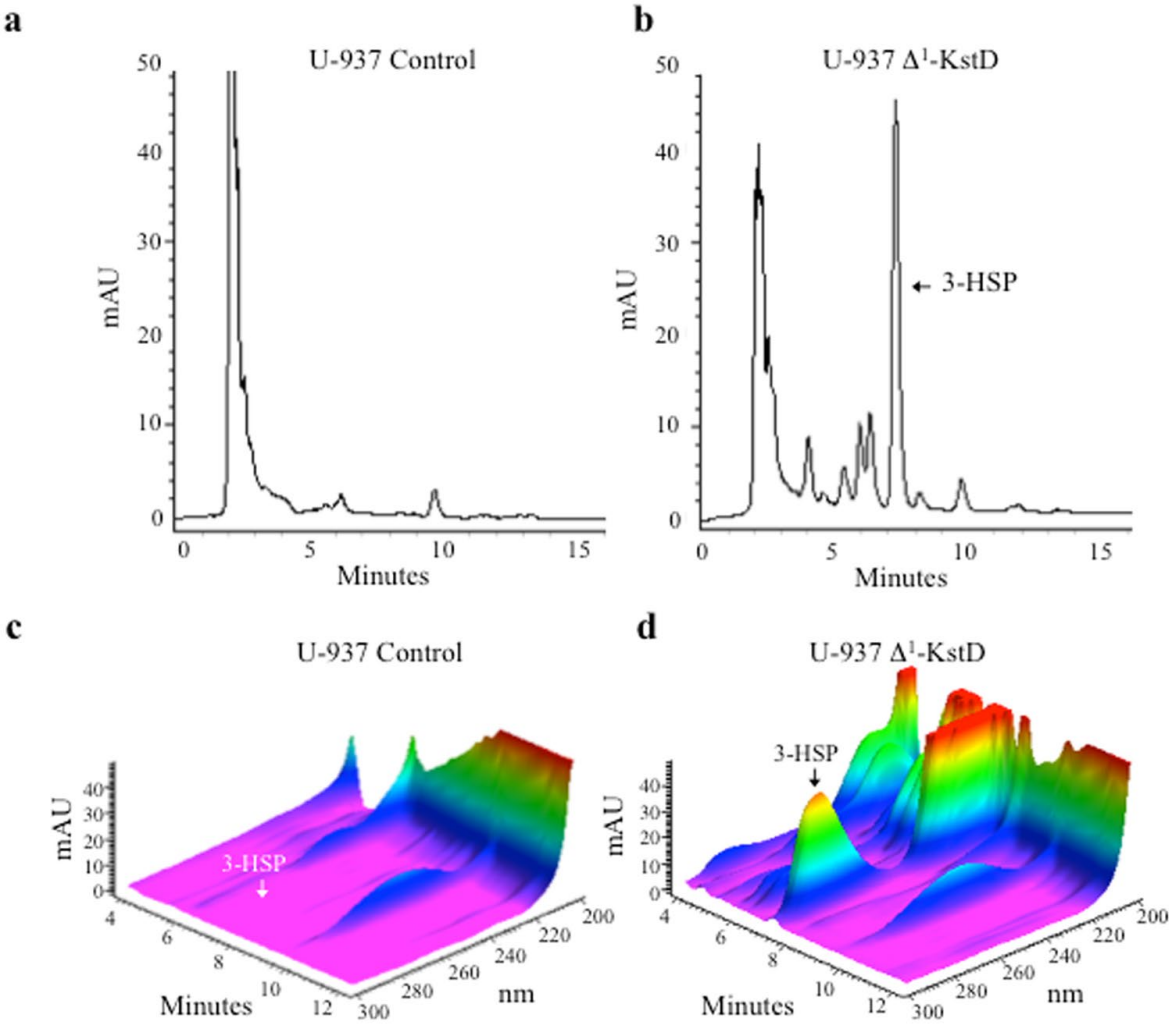
U-937-derived macrophages expressing Δ^1^-KstD catalyze the C-1 and C-2 dehydrogenation of 9-hydroxypregn-4-ene-3,20-dione (9-OHPD) leading to the formation of 3-hydroxy-9,10-secopregn-1,3,5(10)-triene-9,20-dione (3-HSP). Representative HPLC chromatograms (λ_280 nm_) showing the lack of 3-HSP (*t*_*r*_ = 7.2 min) in controls (**a**,**c**) and the presence of 3-HSP (λ_max_: 280 nm; *t*_*r*_ = 7.2 min) generated from extracts of U-937-derived macrophages expressing Δ^1^-KstD (**b**,**d**) after 72 hours showing the accumulation of 3-HSP only in the U-937 cells expressing Δ^1^-KstD. (**c,d**) 3-D chromatogram showing the spectral data (λ_300-200 nm_) plotted against time and absorption (mAU) of the sample run shown in (**a**,**b**), respectively. U-937 controls (left) or U-937 Δ^1^-KstD expressing macrophages (right) were incubated for 72 hours with 17 μg (10 μM) 9-hydroxypregn-4-ene-3,20-dione (9-OHPD, *t*_*r*_ = 5.2 min) produced and isolated from bacterial Kst-9αH lysates.

To prevent CVD, we envisioned two potential paths for future development. One would involve the introduction of a cassette of cholesterol catabolizing enzymes into liver cells, which in theory would generate a shortage of intracellular hepatic cholesterol leading to upregulation of LDL-receptor expression and lower circulating levels of LDLs. A second approach would be to introduce the cholesterol catabolizing cassette of enzymes into patient derived monocytes. After expanding the monocytes in culture, the cholesterol catabolizing cells would be reintroduced into the patient. In theory, the genetically modified monocytes will migrate to inflamed atherosclerotic lesions, differentiate into macrophages, and possibly prevent further plaque formation or even induce the regression of existing plaques by degrading cholesterol in the lesions. For this path, the cholesterol catabolizing enzymes would need to be functional in human monocyte derived macrophages. Therefore, we next conducted studies designed to determine if the cholesterol catabolizing enzymes were functional in human U-937 cells as a surrogate cell line for studying the actions of monocyte derived macrophages. U-937 cells are a widely used experimental model for elucidating mechanisms of human monocyte/macrophage differentiation^47^. Here, we derived stable U-937 monocyte cell lines employing the same lentiviral expression system used to transform the Hep3B cells. The transformed cells were then treated with PMA to induce differentiation into macrophages. The PMA derived macrophage cultures were then treated with various substrates, and catabolites were detected using identical methods to those employed in our studies with Hep3B cells. As observed in the Hep3B cell lines, pregn-1,4-diene-3,20-dione (PDD; t_r_ = 10.0 minutes; λ_max_: 247 nm) and 3-hydroxy-9,10-secopregn-1,3,5(10)-triene-9,17-dione (3-HSP; t_r_ = 7.2 minutes; λ_max_: 280 nm) are only generated in the Δ^1^-KstD_R_-expressing U-937 cells (Figs 7 and 8, respectively). As also observed in Hep3B cells, additional endogenous activity (EA) that was not apparent from the spectral data was identified based on the [^14^C] measured by the in-line scintillation detector in the PD treated U-937 cells (Fig. 7c,d). Currently, the identity of the novel metabolites has not been firmly established. However, based on their decreased retention time (less hydrophobic) they likely reflect the actions of any one of a number of cytochrome P450s that can add a hydroxyl at a position that results in the loss of spectral absorbance between 210–300 nm. As observed in control Hep3B cells, PDD was not detected in U-937 control cells. Together, these studies demonstrate that when expressed in *E. coli* as recombinant humanized enzymes CholD, Δ^1^-KstD, and Kst-9αH have the predicted enzymatic activity. In addition, we show that one of the key missing enzymes (Δ^1^-KstD_R_) can be expressed and has the predicted activity needed for B-ring opening in human Hep3B and U-937 cells.

## Discussion

Our idea to genetically engineer human cells to enable cholesterol catabolism was founded upon a series of unexpected observations made by others studying chronic tuberculosis^48–50^. During the chronic stage of infection, *M*. *tuberculosis* resides intracellularly in macrophages, which allows the bacteria to avoid many host immune responses^51^. When unable to eradicate infection, the host immune system encases the infected macrophages into dense granuloma structures^48^. This restricts the growth of intracellular pathogens, in part, by depriving them of essential nutrients. How *M*. *tuberculosis* survived in phagosomes for extended periods of time was a key unanswered question in the field until surprising observations revealed that, while sequestered in phagosomes, *M*. *tuberculosis* activates operons encoding genes that allow the utilization of host cell cholesterol as a source for carbon and energy^52,53^.

Building upon the pioneering work of others studying cholesterol catabolism in *Mycobacterium tuberculosis*^54^, *Sterolibacterium denitrificans*^55^, *Rhodococcus erythropolis*^43^, and *Rhodococcus rhodochrous*^56^, we decided to test the provocative hypothesis that, via genetic engineering we can introduce synthetic orthologs of bacterial enzymes to enable a cholesterol ring-opening pathway previously lacking in human cells that may prove useful for the removal of surplus cholesterol. The first enzyme required for ring opening is cholesterol-3-OH dehydrogenase (CholD), an NAD(P)^+^ dependent dehydrogenase. CholD oxidizes the 3β-hydroxyl at C-3 of cholesterol (3β-hydroxycholest-5-ene) to yield cholestenone (cholest-4-ene-3-one). Oxidation of the 3β-hydroxyl, producing a ketone at C-3, also results in the isomerization of the double bond between C-5 and C-6 of ring B to C-4 and C-5 of ring A (Fig. 1). The generation of a humanized form of CholD with catalytic activity proved to be remarkably easy. With little more than codon optimization for expression in human cells, a humanized CholD was easily generated (Figs S5, S6, S11). However, it appears that in human cells the activity of CholD cannot go unabated, as stable CholD expressing cell lines were not obtained and when added to cells in culture, cholestenone is relatively toxic to human cells (e.g. LD_50_ = ~75 μM at 72 hours in Hep3B cells; Fig. S15)^57^. This argues that enabling cholesterol catabolism in human cells will require the generation of downstream enzymes with equal or greater catalytic capability.

For the second step in catabolism, we identified two FAD^+^-dependent 3-ketosteroid dehydrogenases (KstD). One is 3-ketosteroid Δ^1^-dehydrogenase (Δ^1^-KstD_R_) from *R*. *erythropolis*. Another is anoxic cholesterol catabolism B enzyme (acmB) from *Sterolibacterium denitrificans* (Δ^1^-KstD_A_). Both enzymes catalyze the desaturation of ring A by introducing a double bond between the C-1 and C-2 atoms of 3-ketosteroid substrates. In bacteria, genetic studies suggested that the side chain cleavage and ring opening pathways were independent^58–61^. The humanized version of *R*. *erythropolis* Δ^1^-KstD (Δ^1^-KstD_R_) demonstrated robust activity against PD and 9-OHPD. However, Δ^1^-KstD_R_ failed to efficiently use CN as a substrate. In contrast, the humanized version of the *S*. *denitrificans’* ortholog Δ^1^-KstD_A_, is active against both CN and PD (Figs S3c–e, S9–S11). To date, the generation of a stable cell line expressing both CholD and Δ^1^-KstD_A_ has not been achieved. This may indicate that the generation of choleste-1,4-diene-3-one (CDN) is toxic to cells. If so, then the last enzyme in the pathway (Kst-9αH) will need robust activity to ensure toxic intermediates do not accumulate, which was not apparent from our studies with Kst-9αH (Fig. S11). Alternatively, the expression of side chain cleavage enzymes may be useful to generate PD, allowing the incorporation of Δ^1^-KstD_R_ into the final cholesterol-catabolizing cassette. Both avenues are currently under further investigation.

## Methods

### Expression Constructs

Details of the cholesterol-3-OH dehydrogenase (CholD), 3-ketosteroid Δ^1^-dehydrogenase (Δ^1^-KstD_R_), anoxic cholesterol metabolism B (Δ^1^-KstD_A_), and 3-ketosteroid-9α-hydroxylase (Kst-9αH) expression construct are provided in Fig. S23–S26, respectively. Briefly, using GeneOptimizer software (GeneArt; Waltham, MA USA), the amino acid sequence encoded by KstD1 from *Rhodococcus erythropolis* (*strain PR4*/NBRC 100887; accession number: C0ZQP5), cholesterol dehydrogenase, gene: I917_07855 from *Mycobacterium tuberculosis* (*strain Haarlem/NITR202*; accession number: R4M4B2), anoxic cholesterol metabolism B (Cholest-4-en-3-one-delta1-dehydrogenase), gene: acmB from *Sterolibacterium denitrificans* (*strain Chol-1st*; accession number: A9XWD7) and kshA5B from *Rhodococcus rhodochrous* (*strain DSM 43269*; KshA5 gene: kshA5, accession number: F1CMY8 and KshB gene: kshB, accession number: F1CMX3) were converted into synthetic expression constructs optimized for *Homo sapiens* codon usage (CholD, Δ^1^-KstD_R_, and Δ^1^-KstD_A_; Tables S1, S2, S3). Kst-9αH was also optimized for expression in *E*. *coli* (Table S4). Flanking Gateway attachment (attB1 and attB2) were added to aid subcloning. A Kozak consensus sequence for translation initiation was added 5′ of the open reading frame for each enzyme. FLAG tags were introduced to aid detection of the recombinant protein following expression in human cells. 5′ of the CholD open reading frame, we added a 6x His tag to aid purification, and a tobacco etch protease recognition site (TEV site) to allow removal of the upstream fusion proteins after purification. A 3′ HA tag was added to Δ^1^-KstD_A_ to aid detection of the recombinant protein after expression.

After DNA synthesis the constructs were ligated into pUC57 (Δ^1^-KstD_R_, GenScript, Piscataway Township, NJ), pMK-RQ (CholD, GeneArt), or pMA-RQ (Δ^1^-KstD_A_ and Kst-9αH, GeneArt). Δ^1^-KstD_R_, CholD, and Δ^1^-KstD_A_ were then subcloned into pBAD-Dest49 for prokaryotic expression as N-terminal HP-Thioredoxin fusion proteins. Kst-9αH was subcloned into pDest14. For expression in eukaryotic cells the constructs were subcloned into pLenti-CMV-Blast, pLenti-CMV-Puro, or pLenti-PGK-Hygro (Addgene #17451, 17452, 19066^62^). Gateway BP and LR reactions were performed following the protocol provided by the manufacturer (Thermo Fisher Scientific) using equimolar concentrations (50 fmols) of DNA. Plasmid DNA was isolated using a Qiagen DNA kit. The fidelity of constructs was verified by DNA sequencing (Laragen; Culver City, CA USA).

### Enzyme Expression

*E*. *coli* (Rosetta2, Novagen; Overexpress C41, Lucigen Corporation) were transformed with pBAD-Dest49-Δ^1^-KstD_R_, pBAD-Dest49-CholD, pBAD-Dest49-Δ^1^-KstD_A,_ or pDest14-Kst-9αH following the protocol provided by the manufacturer. Liquid cultures (1 liter) were incubated at 37 °C (shaking at 250 rpm) until the OD_600_ was 0.4. Arabinose (for pBAD-Dest49 vectors; final concentration (C_f_) = 0.10%) or isopropyl-β-D-thiogalactopyranoside (IPTG; for pDest14 vectors; final concentration (C_f_) = 300 μM) was added to induce expression, and the culture was allowed to grow at 25 °C, shaking at 250 rpm for ~24 hours. The bacteria were collected by centrifugation at 4,000 × g for 20 minutes at 4 °C. The bacterial pellet was weighed and resuspended (1:4; w/v) in chilled lysis buffer (25 mM Tris-HCl, pH 7.5, 500 mM NaCl, 1 mM MgCl_2_, 20 mM imidazole, 1 mM PMSF, 1 × Calbiochem Protease Inhibitor Cocktail Set 1, and 525 U of Pierce Universal Nuclease). Bacteria were lysed by passage (2 times) through a chilled French press (Thermo IEC French Press Cell Disruptor; 18,000 psi). Clarified lysate was obtained by collecting the supernatant generated following centrifugation of the lysate at 28,500 × g for 1 hr at 4 °C.

### Assessment of Enzyme Activity

Because *E*. *coli* do not metabolize sterols, initial assessment of all enzyme activity was made using the clarified lysate generated from *E*. *coli* transformed with either Δ^1^-KstD_R_, CholD, Δ^1^-KstD_A_, Kst-9αH, or a control plasmid. For each assay, 100 μL of clarified lysate was mixed with 100 μM of the indicated substrate (*i*.*e*. 3.87 μg cholesterol (CL) spiked with 20 nCi [^14^C]- labeled CL (Perkin Elmer NEC018250UC, S.A. 50.8 mCi/mmol), 3.87 μg cholestenone (CN), 3.16 μg pregnenolone (PL), or 3.14 μg pregn-4-ene-3,20-dione (PD) spiked with 20 nCi [^14^C]-labeled PD (ARC 1398 A, S.A. 55 mCi/mmol)) in a 2 mL glass HPLC vial for 24 hours with continual rotation. Steroids were then extracted and analyzed by RP-HPLC as described below.

### Steroid Extraction

Samples were extracted with ethyl acetate two times (clarified lysates - 5:1; v/v, partially purified Δ^1^-KstD_R_ - 2.5:1; v/v, and Hep3B or U-937 cells with media - 2:1; v/v), minimizing the interphase between extractions by centrifugation (3,100 × g for 1 minute at 25 °C). The organic phase (containing both substrates and products) from both extractions were pooled and solvent was evaporated under nitrogen. Cholestane analytes were reconstituted in 90% [vol/vol] acetonitrile in H_2_O. Pregnane analytes were reconstituted in acetonitrile:2-propanol:H_2_O (24:16:60). All samples were filtered with Millipore Ultrafree PVDF centrifugal filters (0.1 μm).

### Reverse Phase High Pressure Liquid Chromatography (RP-HPLC) Analysis

Steroids were separated by RP-HPLC using an analytical RP-C18 column (Chromolith 100; end capped; 5 m; 100 by 4.6 mm; Merck, Darmstadt, Germany) and a Hitachi Elite LaChrom HPLC equipped with an in-line Perkin Elmer Radiomatic 150TR flow scintillation analyzer. For separation of cholestane-based analytes, the mobile phase was comprised of a mixture of solvent A (90% [vol/vol] acetonitrile in H_2_O) and solvent B (85% [vol/vol] acetonitrile in 2-propanol). Separation was performed at a flow rate of 1.25 ml min^−1^ at room temperature with an isocratic elution of 100% solvent A from time 0–25 minutes, a linear gradient of 0–100% solvent B from 25–35 minutes, and an isocratic elution of 100% solvent B from 35–45 minutes. For separation of pregnane-based analytes, the mobile phase was comprised of a mixture of solvent C (30% [vol/vol] acetonitrile in H_2_O) and solvent D (80% [vol/vol] 2-propanol in H_2_O). Separation was performed at a flow rate of 0.8 ml min^−1^ at room temperature with a linear gradient starting from 20% to 50% solvent D over 30 minutes.

### Immobilized Metal Affinity Chromatography (IMAC)

Clarified lysate (23.75 mL) from bacteria expressing a humanized N-terminal HP-Thioredoxin Δ^1^-KstD_R_ fusion protein (pBAD-Dest49-Δ^1^-KstD_R_) was loaded onto a nickel-Sepharose column (GE HiTrap Chelating HP column, 1.6 × 2.5 cm; charged with NiSO_4_) and washed with 22 column volumes of 25 mM Tris-HCl, pH 7.5, 500 mM NaCl, and 20 mM imidazole. Δ^1^-KstD_R_ was eluted with imidazole over a linear gradient starting from 5% to 80% buffer B over ten column volumes at a flow rate of 2 mL/min collecting 9 mL fractions at 4 °C using an AKTA FPLC System (GE Healthcare). Buffer A; 25 mM Tris-HCl, pH 7.5, and 500 mM NaCl; buffer B; 25 mM Tris-HCl, pH 7.5, 500 mM NaCl, and 1 M imidazole.

### Nitrotetrazolium Blue Activity Assays

To identify fractions with Δ^1^-KstD_R_ activity, equal volumes (5 μL) of the 9 ml elution fractions generated during IMAC were loaded onto a native PAGE gel (10% acrylamide, 0.24 M Tris-HCl (pH 8.8). Following electrophoresis (50 volts for 5 hours at 4 °C), the gel was incubated in a 40 mL solution of nitrotetrazolium blue (NTB) staining solution (1.24 mg phenazine methylsulfate, 16.4 mg nitrotetrazolium blue, and 1.19 mg pregn-4-ene-3,20-dione in 66.7 mM Tris-buffer) for 5 minutes at 25 °C. Activity was visualized by the generation of an insoluble blue precipitate formed by the reduction of the nitrotetrazolium ring (Fig. S17).

### Assessment of Δ^1^-KstD_R_ Purity

Purity and yield of Δ^1^-KstD_R_ in fractions generated by IMAC were assessed by SDS-PAGE (10% polyacrylamide gels at 50 v for 0.5 hours followed by 125 v for 1.4 hours) followed by Coomassie-blue staining or western analysis. For western analysis, after SDS-PAGE proteins were transferred from the gel to a PVDF membrane in 1X transfer buffer (10 mM CAPS, pH 11, 10% methanol, and 3.4 mM EDTA) using 300 mA for 2.3 hours and then probed with an anti-FLAG antibody (1:1000; Sigma F3163, from mouse). An ECL anti-mouse IgG secondary antibody conjugated to horseradish peroxidase (HRP) (1:10,000, GE Healthcare NA931VS, from sheep) and SuperSignal West Femto Substrate were utilized to visualize antibody binding. Protein concentration was determined using the DC Protein assay kit (Bio Rad) using bovine serum albumin as a standard.

### Resazurin Activity Assays

Fluorometric assays using resazurin were performed at 37 °C in a 96-well format using a BioTek Synergy 2 plate reader (excitation 540 ± 25 nm and emission 620 ± 40 nm). Reactions containing 30 μL of the indicated concentrations of Δ^1^-KstD_R_ (C_f_ = 0.05, 0.19, 0.37, 0.55, 1.1, 1.6, 2.12 nM), 30 μL of 1 mg/mL BSA (C_f_ = 0.1 mg/mL), and 10 μL of 600 μM resazurin (C_f_ = 20 μM) were initiated by the addition of 230 μL of the indicated amount of steroid substrate in 25 mM Tris-HCl, pH 7.5 (V_f_ = 300 μL). Fluorescence measurements were made in each well every 17 seconds for the time indicated. All assays contained a minimum of three replicates and three blanks for baseline subtraction for each condition tested.

### Δ^1^-KstD_R_ Kinetic Analysis (Resazurin Based Assays)

Initial velocities were measured by monitoring the reduction of resazurin at 37 °C. Reaction mixtures containing 0.55 nM, 1.1 nM, or 1.6 nM Δ^1^-KstD_R_ and 0.1 mg/mL BSA were dispensed with a positive displacement syringe (Hamilton: GE health care) prior to initiating the reactions with the addition of the indicated concentrations of steroid substrates (final concentrations: 1, 2.5, 5, 10, 20, 30, and 40 μM) and 20 μM resazurin in 25 mM Tris-HCl, pH 7.5. Fluorescence measurements were made in each well every 17 seconds for a total of 10 minutes. Each concentration measurement consisted of eight replicate wells and four blank wells for baseline subtraction.

### Δ^1^-KstD_R_ Substrate Specificity Assays

Substrate specificity assays (V_f_ = 300 μL) were performed in 25 mM Tris-HCl, pH 7.5 measuring relative fluorescent intensity at 37 °C. Reaction mixtures containing 5.35 nM Δ^1^-KstD_R_ and 0.1 mg/mL BSA were equilibrated for 30 seconds before the reaction was initiated by adding resazurin (C_f_ = 20 μM) along with the indicated substrate (C_f_ = 20 μM) in a 96 well format. Substrates tested: 3β-hydroxypregn-5-en-20-one (pregnenolone) (Sigma), pregn-4-ene-3,20-dione (progesterone) (Sigma), (11β)−11,17,21-trihydroxypregna-1,4-diene-3,20-dione (prednisolone) (Sigma), 4-pregnen-17-ol-3,20-dione (17-hydroxyprogesterone) (Steraloids Q3360), 4-pregnen-21-ol-3,20-dione (11-deoxycorticosterone) (Steraloids Q3460), (11β)-11,21-dihydroxypregn-4ene-3,20-dione (corticosterone) (Sigma C-2505), (11β)-11,17,21-trihydroxypregn-4-ene-3,20-dione (hydrocortisone) (Sigma No. H-4001), 17α,21-dihydroxy-4-pregnene-3,11,20-trione (cortisone) (Sigma C-2755), 11β,21-dihydroxy-3,20-dioxopregn-4-en-18-al (aldosterone) (Acros Organics 215360050), 11β,17α,21-trihydroxy-4-pregnene-3,20-dione 21-hemisuccinate sodium salt (hydrocortisone 21-hemisuccinate) (Sigma), 5α-androstan-3α-ol-17-one (androsterone) (Steraloids A2420), 5-androsten-3β-ol-17-one (DHEA/dehydroepiandrosterone) (Steraloids A8500), 5α-androstan-17β-ol-3-one (5α-DHT) (Steraloids A2570), 4-androsten-17β-ol-3-one (testosterone) (Steraloids A6950), 4-androsten-3,17-dione (androstenedione) (Steraloids A6030), 17β-hydroxy-4-androsten-3-one 17-enanthate (testosterone enanthate) (Sigma**)**, 3β-hydroxy-5-cholestene (cholesterol) (Sigma), 5-cholesten-3-one (cholestenone) (Sigma), 11β-(4-dimethyl-amino)-phenyl-17β-hydroxy-17-(1-propynyl)-estra-4,9-dien-3-one (mifepristone) (Roussel UCLAF 7 A 4087 RU 38486), 7α-acetylthio-3-oxo-17α-pregn-4-ene-21,17-carbolactone (spironolactone) (Sigma), and 4-cholesten-7β-ol-3-one (7β-hydroxycholestenone) (Steraloids C6230-000). Unless indicated otherwise, for each experiment four replicates and four blanks for baseline subtraction were made for each substrate.

### Cell Culture

Hep3B (ATCC HB-8064) and HEK293FT (Thermo Fisher R70007) cells were grown in DMEM culture media containing 1 mM sodium pyruvate, 0.5x NEAA, and 10% fetal bovine serum at 37 °C and 5% CO_2_. U-937 cells (ATCC CRL-1593.2) were cultured in RPMI-1640 media (Gibco) supplemented with 1× non-essential amino acids, 100 units/mL penicillin, 100 μg streptomycin, and 10% fetal bovine serum. To differentiate U-937 monocytes into macrophages, cells were seeded (6 × 10^6^ cells) into 100 mm dishes coated with 0.1% gelatin. Phorbol 12-myristate 13-acetate (PMA P1585, 0.1 mg/mL in DMSO) was added at a final concentration of 200 nM for 48 hours. Following PMA treatment, media was removed, cells rinsed twice with PBS, and allowed to continue differentiating for 72 hours.

### Lentiviral Packaging

Lentiviral particles encoding the Δ^1^-KstD_R_ constructs were produced with HEK293FT cells using the third-generation lentiviral packaging system (Addgene #s: 12251, 12253, and 12259). Packaging vectors (7.5 μg pMDLg/pRRE, 3.75 μg RSV-REV, and 4.5 μg PMD2.G) and each transfer vector (3 μg pLenti-CMV-Blast (706-1)-Δ^1^-KstD_R_, pLenti-CMV-Puro (w118-1)-Δ^1^-KstD_R_, or pLenti-PGK-Hygro-(w530-1)- Δ^1^-KstD_R_) were diluted with 1.875 mL Opti-MEM (1 μg plasmid DNA/100 μL Opti-MEM) in a glass vial. DNA was mixed gently by tapping bottom of vial 30 times. For transfections using XtremeGene HP, a 2:1 (wt:vol) ratio of DNA to XtremeGene HP (37.5 μL XtremeGene HP) was used. For transfections using XtremeGene 9, a 3:1 ratio (wt:vol) of DNA to XtremeGene 9 (56.25 μL XtremeGene 9) was used. The transfection reagent was added to the glass vial, mixed gently by tapping 30 times, incubated at room temperature for 30 minutes, added to 12 mL of media, mixed by inversion and added slowly to the side of a 0.10% gelatin coated T75 flask containing HEK293FT cells that were pre-seeded (2 × 10^6^ cells) and grown to ~70% confluencey. The flask was slowly laid flat to minimize disturbing the monolayer of cells. Cells were allowed to produce lentiviral particles for 48 hours. Following incubation, the supernatant containing the viral particles was removed, subjected to centrifugation (1,625 × g) for 2 minutes, filtered through a 0.45 μm polyethersulfone membrane using a syringe, separated into 500 μL aliquots, flash frozen in a dry ice/ethanol bath, and stored at −80 °C until use.

### Stable Expression of Δ^1^-KstD_R_ in Hep3B and U-937 Cells

Hep3B cells (2.3 × 10^5^ cells) were grown to ~70% confluencey in 60 mm dishes containing 5 mL of media. U-937 monocytes (1.0 × 10^5^ cells/mL) were seeded in T25 flasks containing 5 mL media. Cells were transduced with 0.5 mL of the total 13 mL of the viral supernatant. Cells were allowed to incubate with the viral supernatant for 48 hours prior to selection with the appropriate antibiotic for two weeks (0.05 mg/mL hygromycin for pLenti-PGK-Hygro-(w530-1)-Δ^1^-KstD_R_, 0.001 mg/mL puromycin for pLenti-CMV-Puro-(w118-1)-Δ^1^-KstD_R_, or 0.012 mg/mL blasticidin for pLenti-CMV-Blast-(706-1)-Δ^1^-KstD_R_).

### SDS-PAGE and Western Blot of Eukaryotic Cell Lines

Hep3B cells were grown in 60 mm dishes, washed with PBS (2x), and collected by scraping in 500 μL RIPA buffer (10 mM Tris-CL, pH 8.0, 1 mM EDTA, 0.5 mM EGTA, 1% Triton X-100, 0.1% sodium deoxycholate, 0.1% SDS, 140 mM NaCl, and 1 mM PMSF). U-937 cells were collected by centrifugation, washed with PBS, and resuspended in 500 μL RIPA buffer. Cells were mechanically lysed on ice using a syringe with a 27-gauge needle. Protein samples were mixed with an equal volume of 2x Laemmli SDS-sample buffer, placed in near boiling water for 5 minutes, and subjected to centrifugation at 15,000 x g for 10 minutes at 4 °C. Protein samples (25 μg) were separated using SDS-PAGE on a 10% polyacrylamide gel, transferred to a PVDF membrane, and probed with anti-FLAG (1:1000; Millipore MAB3118, from mouse). ECL anti-mouse IgG secondary antibody conjugated to HRP (1:10,000 GE Healthcare NA931VS, from sheep) and SuperSignal West Femto Substrate were used for visualization.

### Enzyme Activity Assessment of Stable Hep3B and U-937 Cells Lines

Hep3B cells stably expressing the indicated expression construct were seeded (2.3 × 10^5^ cells) into 60 mm dishes and grown until confluent. U-937 monocytes were differentiated into macrophages as described above. At time 0, the media was removed and the cells were washed twice with PBS. New serum free culture media (Hep3B) or RPMI-1640 supplemented with 2% FBS (U-937) and the indicated substrate (10 μM) were added to cells and incubated at 37 °C. At the indicated points in time, the cells were scraped in cultured media and the analytes were extracted and analyzed by RP-HPLC as described above.

### 9-Hydroxypregn-4-ene-3,20-dione (9-OHPD) Production and Isolation

To generate 9-hydroxypregn-4-ene-3,20-dione (9-OHPD), 7 mL of clarified lysate from bacteria expressing Kst-9αH (3-ketosteroid-9α-hydroxylase), 1.25 mg pregn-4-ene-3,20-dione (PD), and 70 μM NADH was incubated for 48 hours at 25 °C with continual rotation. The reaction was stopped and extracted using ethyl acetate (2:1; v/v), thrice. RP-HPLC analysis revealed ~ 76.6% conversion of PD to 9-OHPD (λ_maxmax_ 245 nm; t_r_ = 5.2 min). The analytes were dried under nitrogen, resuspended in ethanol, and stored as a 5.8 mM 9-OHPD stock solution at 4 °C.

### LC-MS/MS Analysis

Liquid chromatography-tandem mass spectrometry (LC-MS/MS) analyses were performed with an Agilent 1200 series HPLC coupled to a Thermo LTQ-Orbitrap XL mass spectrometer. A Waters XBridge C18 reverse phase analytical column (3.5 µM, 1.0 × 150 mm, P/N 186003606) was used to achieve chromatographic separation between PD and PDD. A binary solvent system was used with solvent A consisting of 3.0% acetonitrile in H_2_O and solvent B consisting of 3.0% H_2_O in acetonitrile, both containing 0.2% formic acid. A flow rate of 40 µl per minute and a linear solvent gradient was used to elute the samples from the column starting at 40% B and slowly ramping to 90% B over a period of 18 minutes. The solvent was held at 90% B for 5 minutes before returning to 40% B at the end of the run, with a total run time of 30 minutes. Electrospray ionization was used to introduce the sample into the mass spec using the Thermo HESI source with positive polarity and a voltage of 4.0 kV. One full scan from 200–800 m/z was performed at 60,000 resolution in the FTMS, followed by data dependent MS/MS scans in the linear ion trap of the top most intense ions noted in a parent mass list, which included the labeled and unlabeled parent m/z values for PD [^13^C_3_^12^C_18_H_30_O_2_ (318.2426 m/z), C_21_H_30_O_2_ (315.2324 m/z)] and PDD [^13^C_3_^12^C_18_H_28_O_2_ (316.2269 m/z), C_21_H_28_O_2_ (313.2168 m/z)]. An injection volume of 6.0 µl was used for each sample, with blanks run in-between each sample to minimize carryover. Both the labeled and unlabeled PD and PDD were observed from the *E*. *coli* and Hep3B cell lysates with MS2 fragmentation for confirmation. All masses were measured within 5.0 ppm mass accuracy.

## Supplementary information

supplemental information

## Data Availability

The authors declare that data, associated protocols and materials will be made available to others without undue qualifications in material transfer agreements.
