# Supplementary material for: Development of a Synthetic 3-ketosteroid Δ1-dehydrogenase for the Generation of a Novel Catabolic Pathway Enabling Cholesterol Degradation in Human Cells
Source: Sci Rep. 2019 Apr 12;9:5969. doi: 10.1038/s41598-019-42046-8 (PMC6461610; doi:10.1038/s41598-019-42046-8)
Supplement: Supplementary file 1 — supplemental information [file 41598_2019_42046_MOESM1_ESM.pdf]

Supplementary Information

Development of a Synthetic 3-ketosteroid-Δ[1]-dehydrogenase for the Generation of a Novel Catabolic Pathway Enabling Cholesterol Degradation in Human Cells

Brandon M. D'Arcy[1,2], Mark R. Swingle[1], Lindsay Schambeau[2], Lewis Pannell[2], Aishwarya Prakash[2], Richard E. Honkanen[1,2*]

From the
[1]Department of Biochemistry & Molecular Biology, University of South Alabama, Mobile, AL 36688;

[2]Mitchell Cancer Institute, 1660 Springhill Ave, Mobile, AL 36604

Running title: 3-ketosteroid Δ[1]-dehydrogenase is active in human cells

*Corresponding author:
Richard E. Honkanen: Department of Biochemistry & Molecular Biology, University of South Alabama, Mobile, AL 36688;
rhonkanen@southalabama.edu; Tel. (251)-460-6859

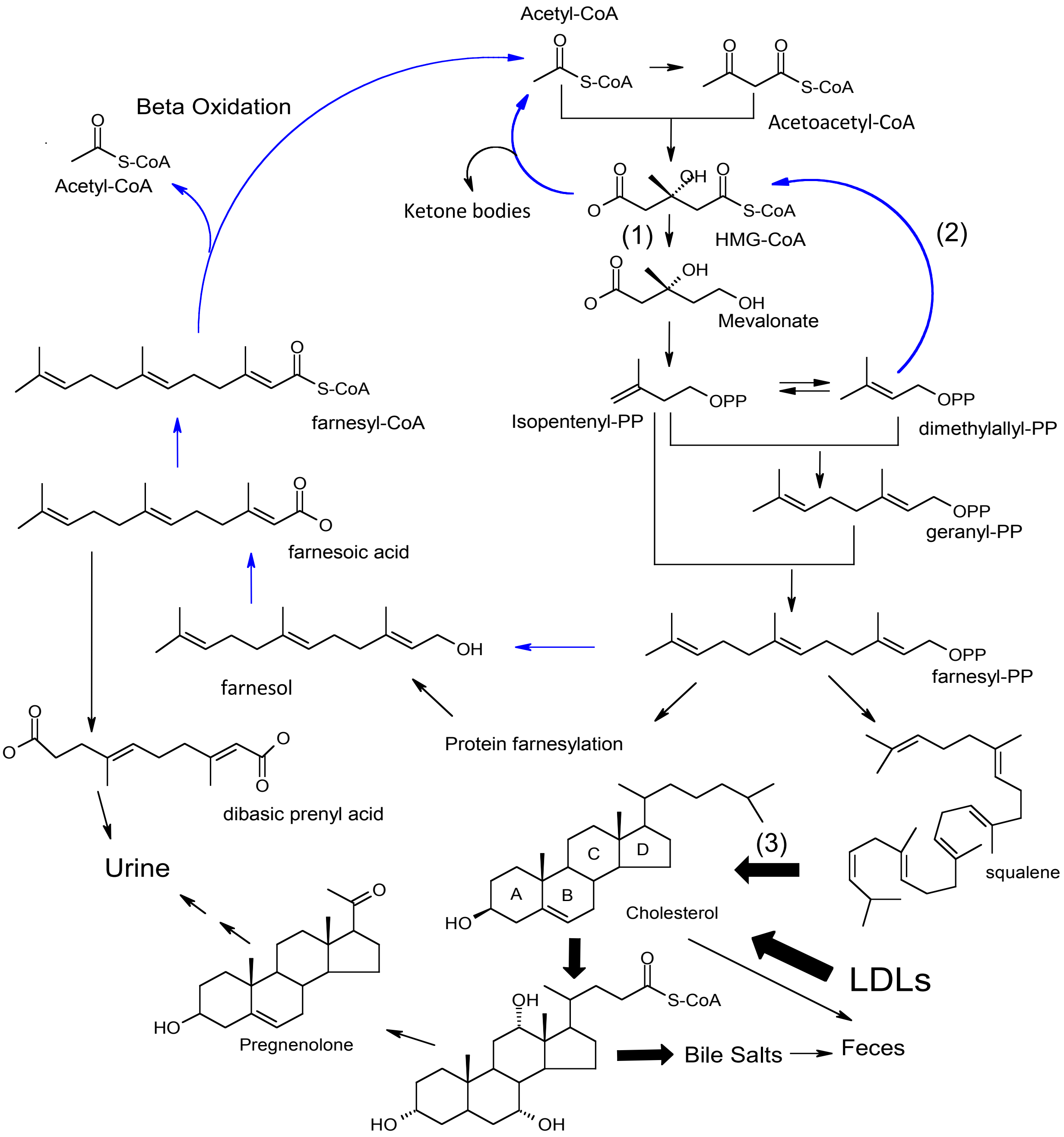

Figure S1. Summary of human cholesterol synthesis and metabolism. All carbons used in cholesterol synthesis are derived from acetyl-CoA (from the catabolism of ketogenic amino acids and beta-oxidation of fatty acids). The reduction of 3-hydroxy-3-methylglutaryl-CoA (HMG-CoA) to mevalonate (1) was originally believed to represent the first irreversible step in cholesterol synthesis. However, more recent studies have revealed a trans-methylglutaconate shunt (2), converting dimethylallyl pyrophosphate (PP) back into HMG-CoA. In cholesterol synthesis dimethylallyl-PP and isopentenyl-PP are converted to geranyl-PP via head to tail condensation, and farnesyl-PP is produced by the condensation of geranyl-PP and a second isopentenyl-PP. Farnesyl-PP is used for protein modification (protein-farnesylation), and in a head to head condensation reaction two molecules are used to produce squalene. In humans, the conversion of squalene to cholesterol occurs in several steps (3), and after ring closure (squalene-2,3-oxide cyclization to generate lanosterol), humans lack the enzymes necessary to hydrolyze the cholestane ring. Thus, endogenous conversion back to farnesol is not possible. Still, humans express many enzymes that can modify aspects of the cholestane ring and C-17 side chain.

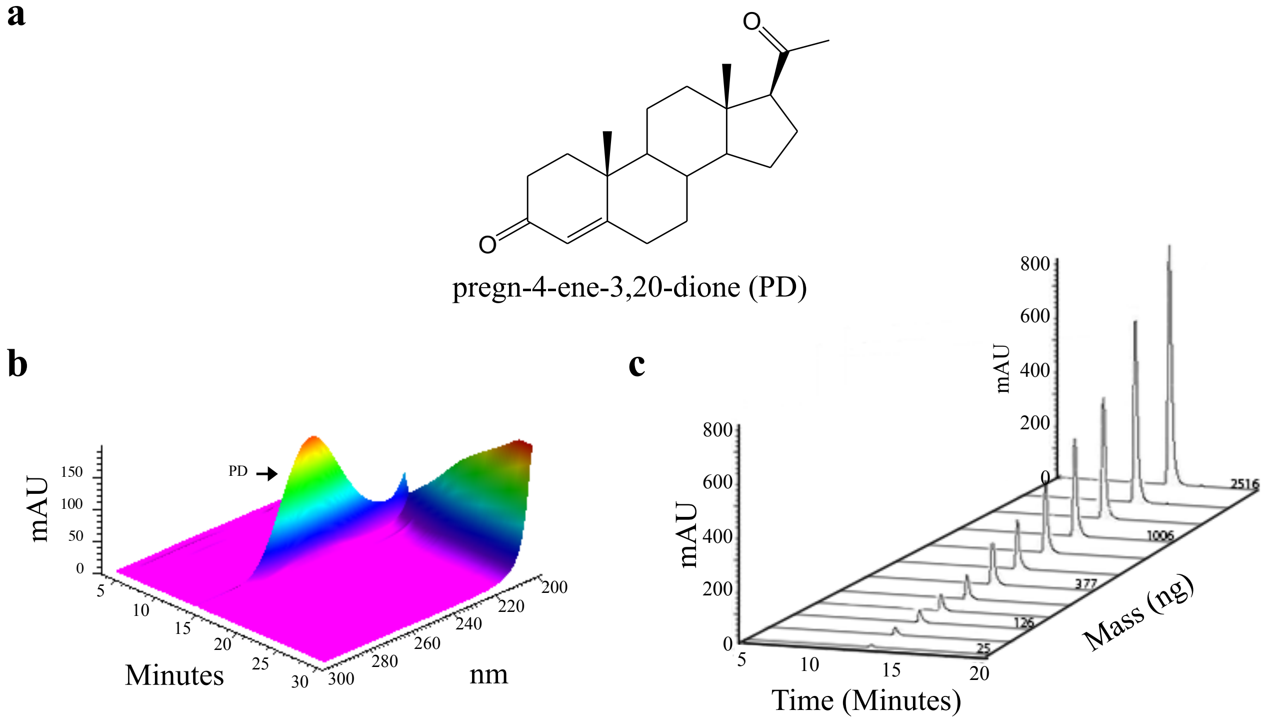

d

Mass (μg)	AUC
0.025	385193
0.076	1144434
0.129	1932149
0.182	2726643
0.257	3850619
0.391	5851692
0.523	7824425
0.788	11772883
1.050	15689153
1.315	19632318
1.979	29545695
2.648	39529936

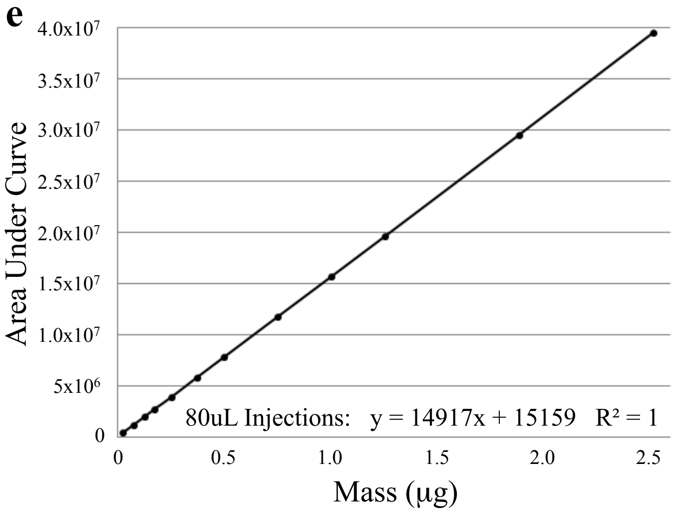

Figure S2. Demonstration of the sensitivity and quantitative ability in assessing the number of micrograms in a serial dilution of pregn-4-ene-3,20-dione (PD) by RP-HPLC. (a) Chemical structure of PD. (b) 3-D chromatogram of PD (λ_{max}: 245 nm; t_r = 13.8 min) showing spectral data ($\lambda_{300\text{-}200\ nm}$) plotted against time and absorption (mAU). (c) Serial 2-D chromatograms with increasing concentrations of PD. (d, e) Injection volumes of 80 μL from serial dilutions between 0.025 μg to 2.5 μg PD resulted in a calibration curve with an R^2 of 1.

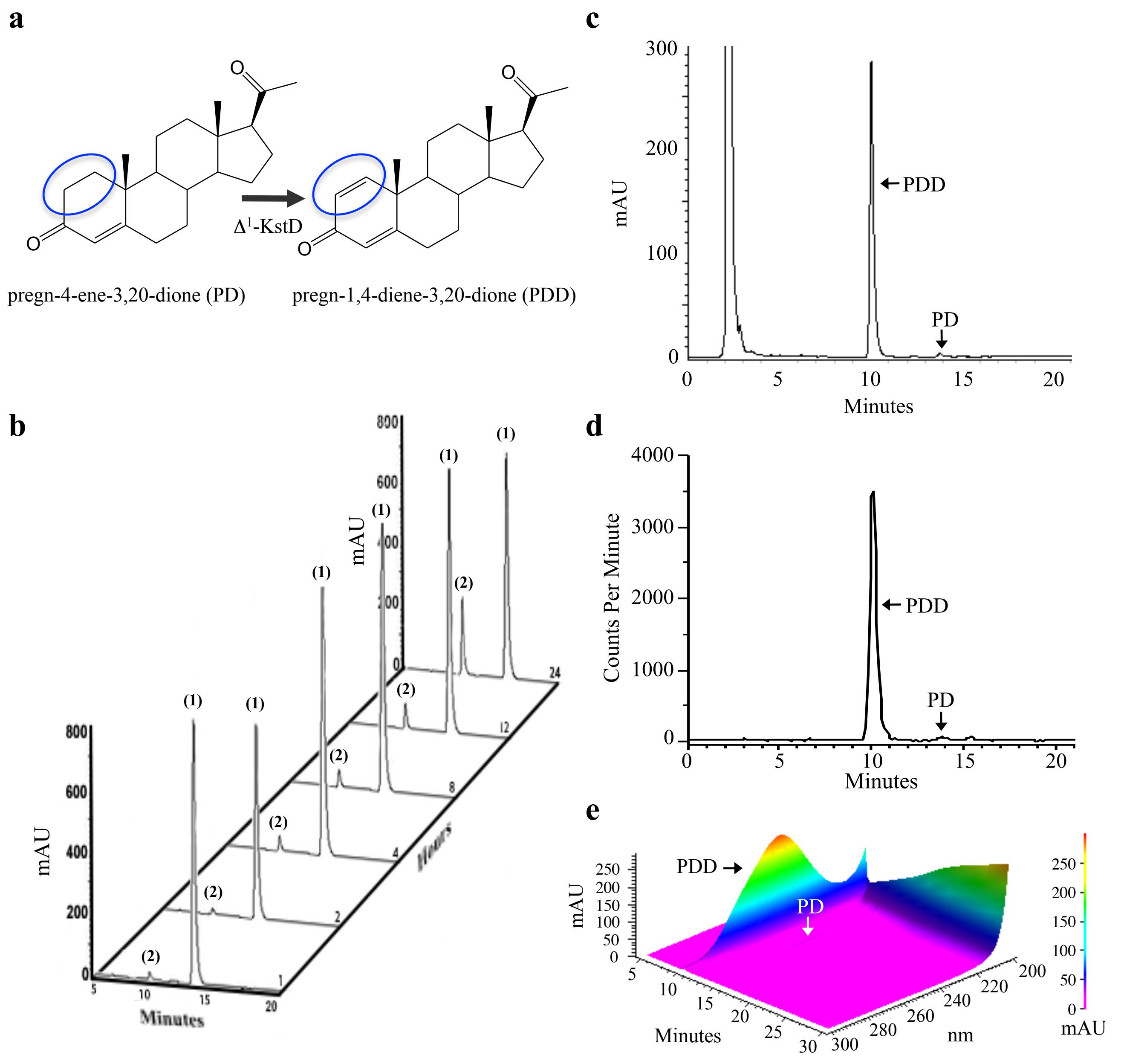

Figure S3. Humanized 3-ketosteroid Δ^1-dehydrogenases (Δ^1-KstD$_R$ and Δ^1-KstD$_A$) catalyze the C-1 and C-2 dehydrogenation of pregn-4-ene-3,20-dione (PD) producing pregn-1,4-diene-3,20-dione (PDD) in a time-dependent manner. (a) Structures and the reaction summary. (b) Representative RP-HPLC chromatograms obtained from clarified bacterial lysates expressing Δ^1-KstD$_R$ incubated with 159 μg of pregn-4-ene-3,20-dione for 24 hours. The chromatograms show a time-dependent (1, 2, 4, 8, 12, and 24 hours) increase in the product (PDD; λ_{max}: 247 nm; t_r = 10.0 min) with a concomitant decrease in the substrate (PD; λ_{max}: 245 nm; t_r = 13.8 min). (c) Representative RP-HPLC chromatogram showing the complete conversion of PD (λ_{max}: 245 nm; t_r = 13.8 min) to PDD (λ_{max}: 247 nm; t_r = 10.0 min) generated by the incubation of C4-[^{14}C] PD with clarified bacterial lysates of *E. coli* expressing Δ^1-KstD$_A$ incubated with for 24 hours. (d) [^{14}C] measured by the in-line scintillation detector corresponding to the chromatogram show in (c). (e) 3-D chromatogram showing the spectral data ($\lambda_{300\text{-}200\ nm}$) plotted against time and absorption (mAU) of the sample run in (c). For all experiments shown, lipids were extracted and analyzed by RP-HPLC as described in the methods.

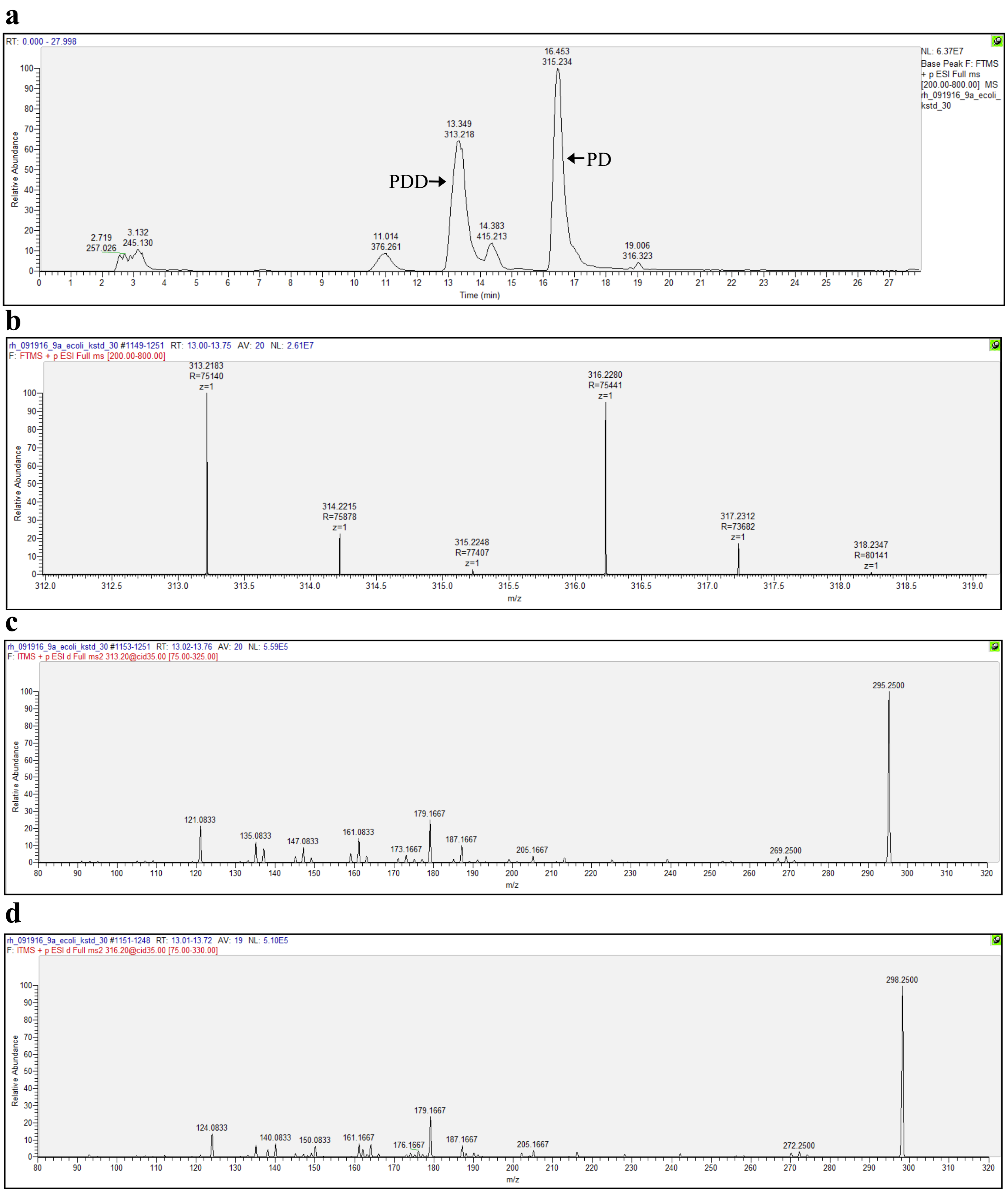

Figure S4. Mass spectrometry analysis confirming the production of pregn-1,4-diene-3,20-dione (PDD) from pregn-4-ene-3,20-dione (PD). (a) Representative RP-HPLC chromatogram generated from clarified bacterial lysates of *E. coli* expressing Δ^1-KstD$_R$ following incubation with 50 µM PD and 50 µM C2,3,4-^{13}C-PD (t_r = 16.45 min) for 24 hours. (b) MS1 spectrum of PDD (t_r = 13.35 min) in panel (a) reveals two peaks representing unlabeled PDD (313.2183 m/z) and labeled PDD (316.2280 m/z). (c) MS2 spectrum of unlabeled PDD in panel (b). (d) MS2 spectrum of labeled PDD in panel (b).

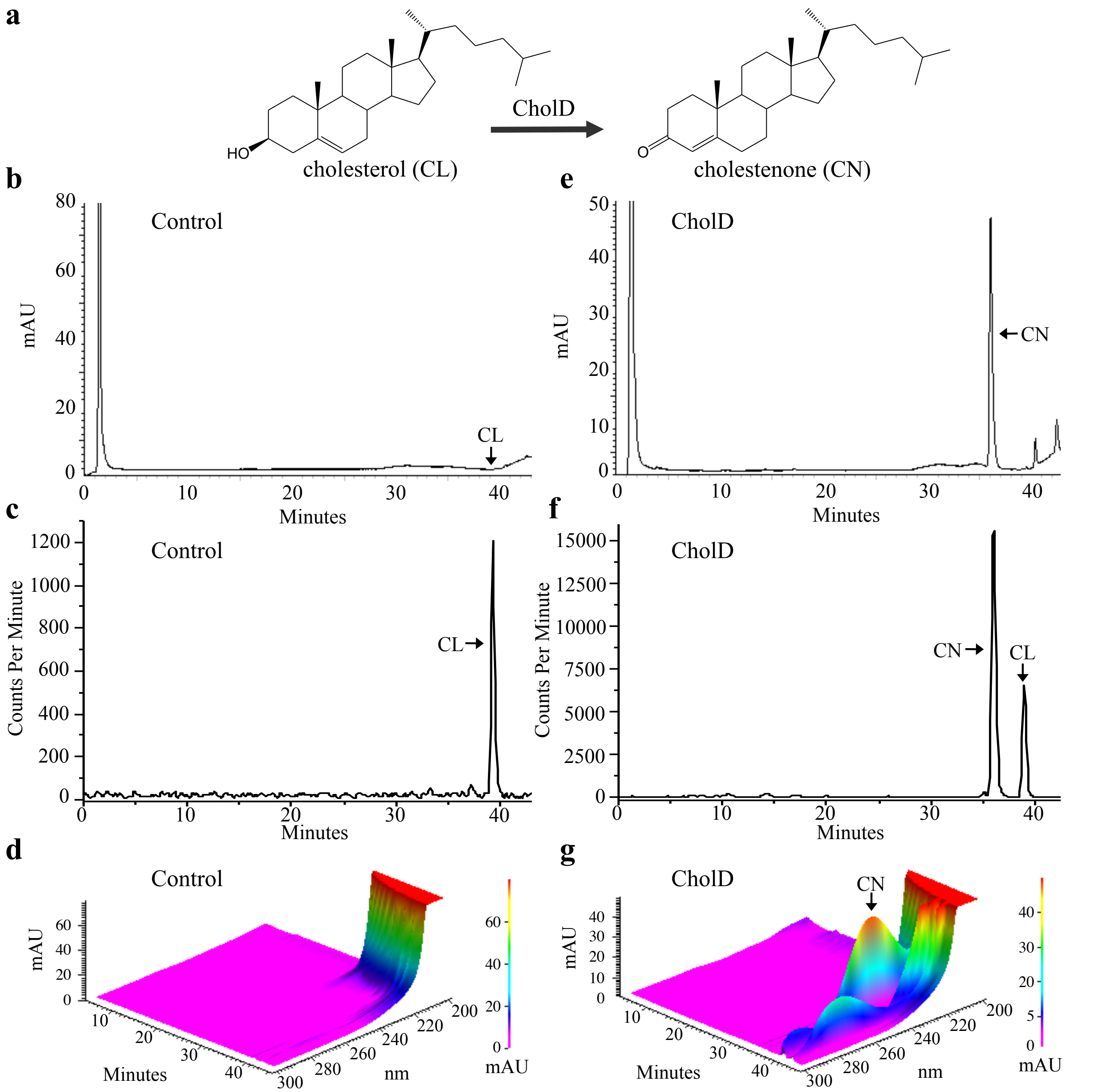

Figure S5. Humanized cholesterol-3-OH dehydrogenase (CholD) oxidizes the 3β-hydroxyl of cholesterol (CL) to produce cholestenone (CN). Clarified lysates produced in an identical manner from either *E. coli* expressing CholD or *E. coli* transformed with an empty (pUC19) expression plasmid (control) were incubated with C4-[^{14}C] labeled CL for 24 hours. Following incubation, lipids were extracted and analyzed by RP-HPLC as described in the methods. (a) Chemical structures and reaction overview. (b) Representative HPLC chromatogram of the 24 hour time point showing only CL (λ_{max}: < 200 nm; t_r = 38.9 min) in control extracts. (c) [^{14}C] measured by in-line scintillation detector corresponding to the chromatogram shown in (b). (d) 3-D chromatogram showing the spectral data ($\lambda_{300\text{-}200\ nm}$) plotted against time and absorption (mAU) of the sample run in (b). (e) Representative HPLC chromatogram of the 24 hour time point showing CL (λ_{max}: < 200 nm; t_r = 38.9 min) and CN (λ_{max}: 239 nm; t_r = 36.9 min) in samples from *E. coli* expressing CholD treated in an identical manner as controls. (f) [^{14}C] measured by in-line scintillation detector corresponding to the chromatogram shown in (e). (g) 3-D chromatogram showing the spectral data ($\lambda_{300\text{-}200\ nm}$) plotted against time and absorption (mAU) of the sample run in (e).

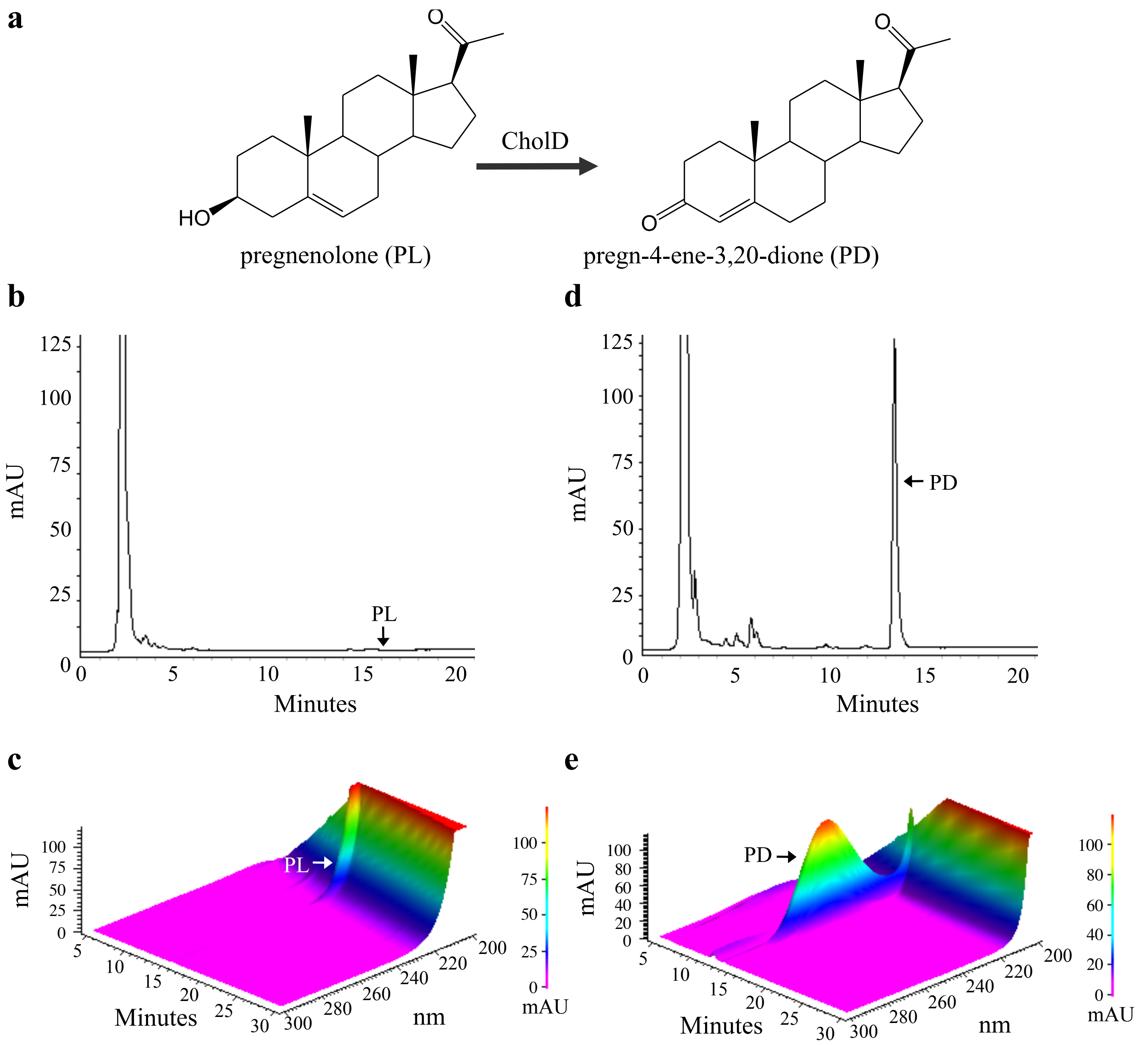

Figure S6. Humanized cholesterol-3-OH dehydrogenase (CholD) oxidizes the 3β-hydroxyl of pregnenolone (PL) to produce pregn-4-ene-3,20-dione (PD). Clarified lysates produced in an identical manner from either *E. coli* expressing CholD or *E. coli* transformed with an empty (pUC19) expression plasmid (control) were incubated with PL for 24 hours. Following incubation, lipids were extracted and analyzed by RP-HPLC as described in the methods. (a) Chemical structures and reaction overview. (b) Representative HPLC chromatogram of the 24 hour time point showing only PL (λ_{max}: < 200 nm; t_r = 15.5 min) in control extracts. (c) 3-D chromatogram showing the spectral data ($\lambda_{300\text{-}200\ nm}$) plotted against time and absorption (mAU) of the sample run in (b). (d) Representative HPLC chromatogram of the 24 hour time point showing PD (λ_{max}: 245 nm; t_r = 13.8 min) in samples from *E. coli* expressing CholD treated in an identical manner as controls. (e) 3-D chromatogram showing the spectral data ($\lambda_{300\text{-}200\ nm}$) plotted against time and absorption (mAU) of the sample run in (d).

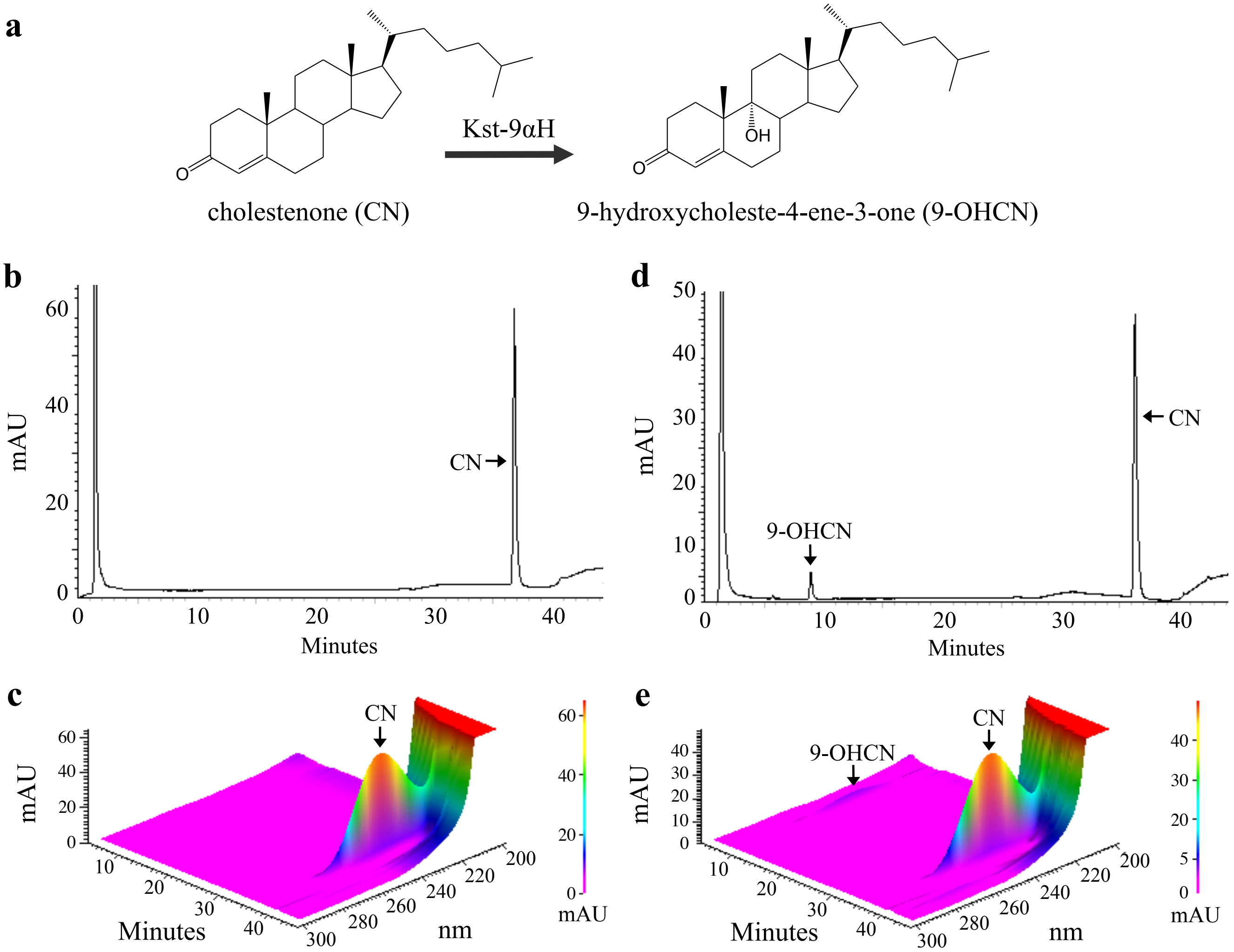

Figure S7. 3-ketosteroid-9α-hydroxylase (Kst-9αH) hydroxylates C-9 of cholestenone (CN) to produce 9-hydroxycholeste-4-ene-3-one (9-OHCN). Clarified lysates produced in an identical manner from either *E. coli* expressing Kst-9αH or *E. coli* transformed with an empty (pUC19) expression plasmid (control) were incubated with CN for 24 hours. Following incubation, lipids were extracted and analyzed by RP-HPLC as described in the methods. (a) Chemical structures and reaction overview. (b) Representative HPLC chromatogram of the 24 hour time point showing only CN (λ_{max}: 239 nm; t_r = 36.9 min) in control extracts. (c) 3-D chromatogram showing the spectral data ($\lambda_{300\text{-}200\ nm}$) plotted against time and absorption (mAU) of the sample run in (b). (d) Representative HPLC chromatogram of the 24 hour time point showing 9-OHCN (λ_{max}: 239 nm; t_r = 8.9 min) in samples from *E. coli* expressing Kst-9αH treated in an identical manner as controls. (e) 3-D chromatogram showing the spectral data ($\lambda_{300\text{-}200\ nm}$) plotted against time and absorption (mAU) of the sample run in (d).

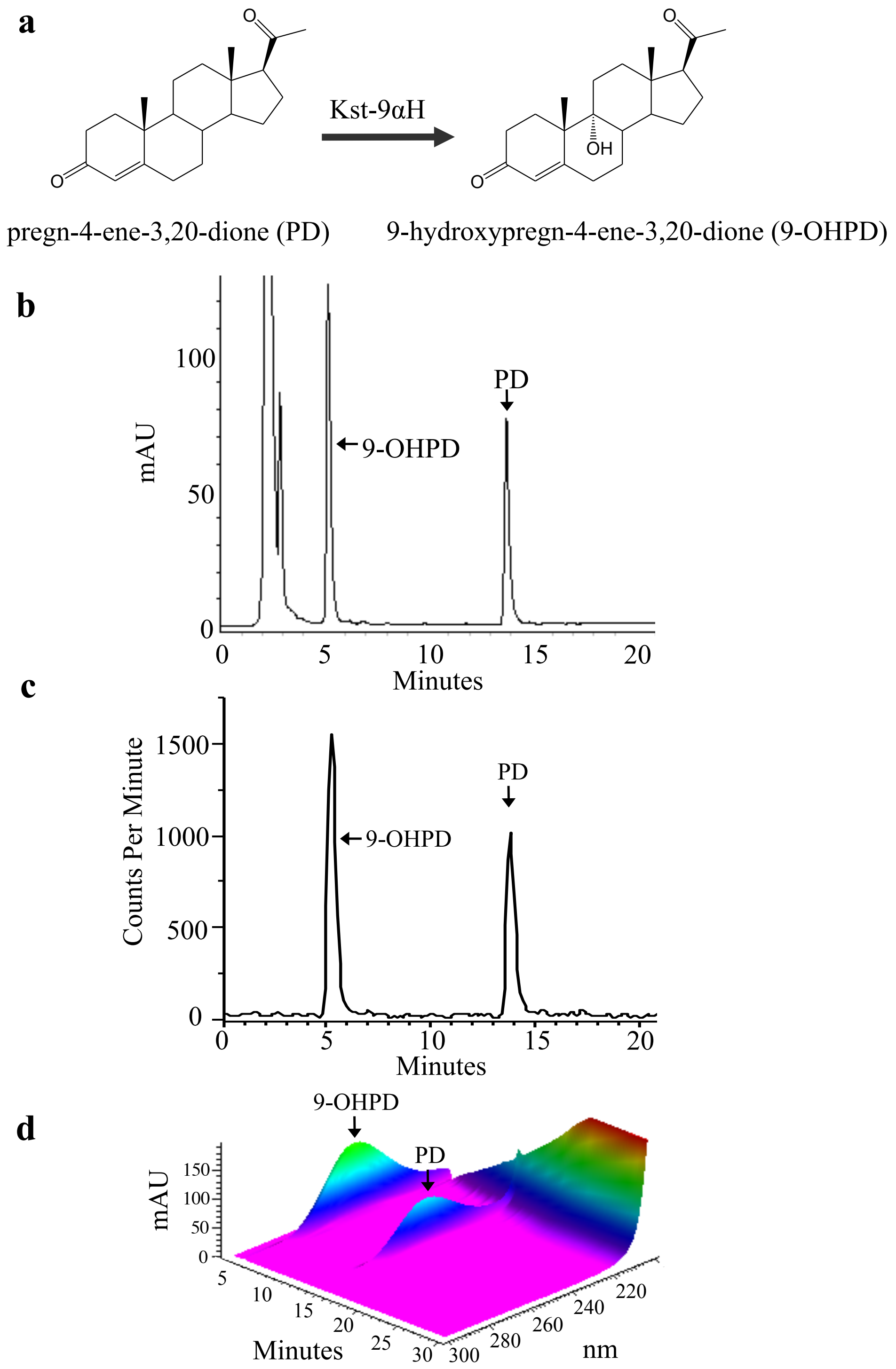

Figure S8. 3-ketosteroid 9α-hydroxylase (Kst-9αH) hydroxylates C-9 of pregn-4-ene-3,20-dione (PD) generating 9-hydroxypregn-4-ene-3,20-dione (9-OHPD). Clarified lysate from *E. coli* expressing Kst-9αH was incubated with C4-[^{14}C] labeled PD for 24 hours. Following incubation, lipids were extracted and analyzed by RP-HPLC as described in the methods. (a) Chemical structures and reaction overview. (b) Representative HPLC chromatogram of the 24 hour time point showing 9-OHPD (λ_{max}: 245 nm; t_r = 5.2 min) in a sample from *E. coli* expressing Kst-9αH. (c) [^{14}C] measured by in-line scintillation detector corresponding to the chromatogram shown in (b). (d) 3-D chromatogram showing the spectral data ($\lambda_{300\text{-}200\ nm}$) plotted against time and absorption (mAU) of the sample run in (b).

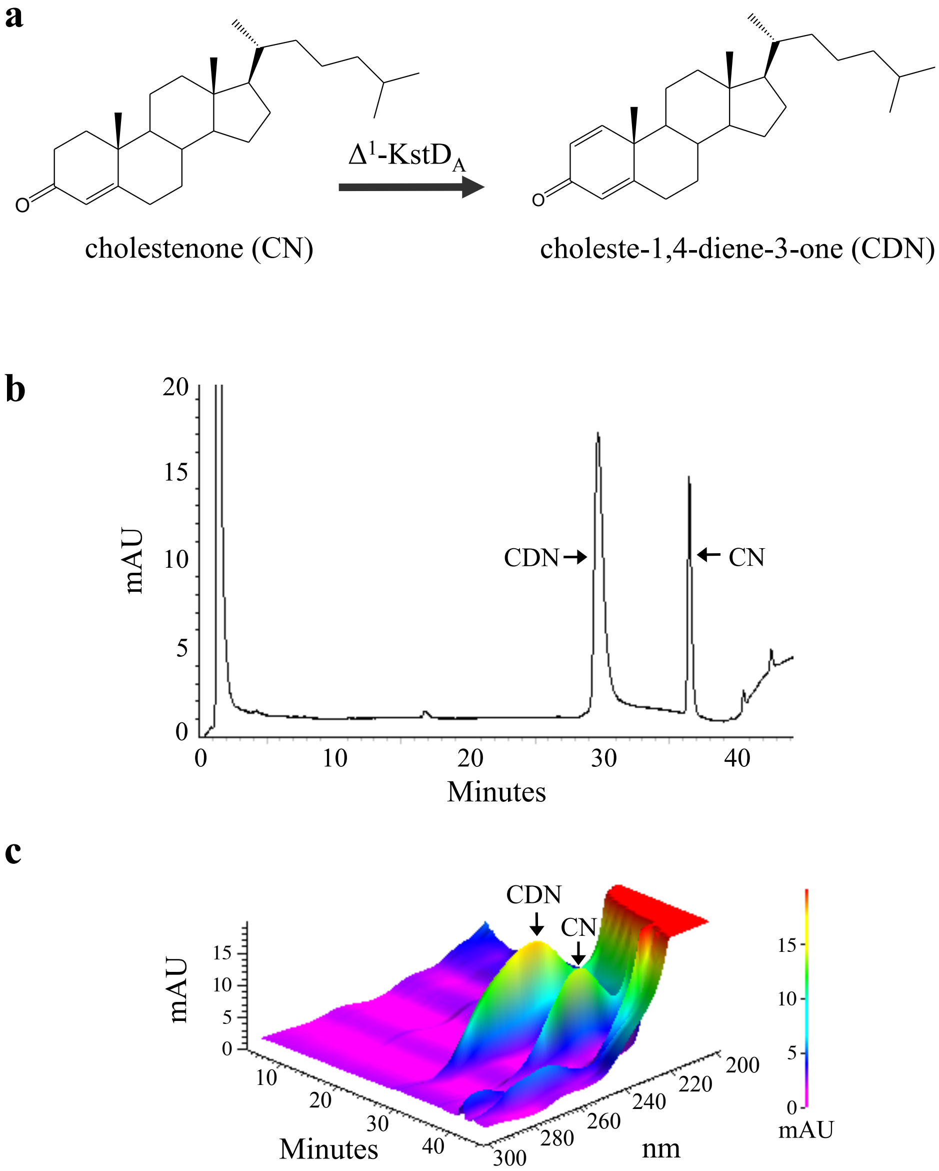

Figure S9. Humanized Δ^1-KstD$_A$ is a second 3-ketosteroid Δ^1-dehydrogenase that catalyzes the C-1 and C-2 dehydrogenation of cholestenone (CN) producing choleste-1,4-diene-3-one (CDN). Clarified lysate from *E. coli* expressing Δ^1-KstD$_A$ was incubated with CN for 24 hours. Following incubation, lipids were extracted and analyzed by RP-HPLC as described in the methods. (a) Chemical structures and reaction overview. (b) Representative HPLC chromatogram of the 24 hour time point showing CDN (λ_{max}: 241 nm; t_r = 29.8 min) in a sample from *E. coli* expressing Δ^1-KstD$_A$. (c) 3-D chromatogram showing the spectral data ($\lambda_{300\text{-}200\ nm}$) plotted against time and absorption (mAU) of the sample run in (b).

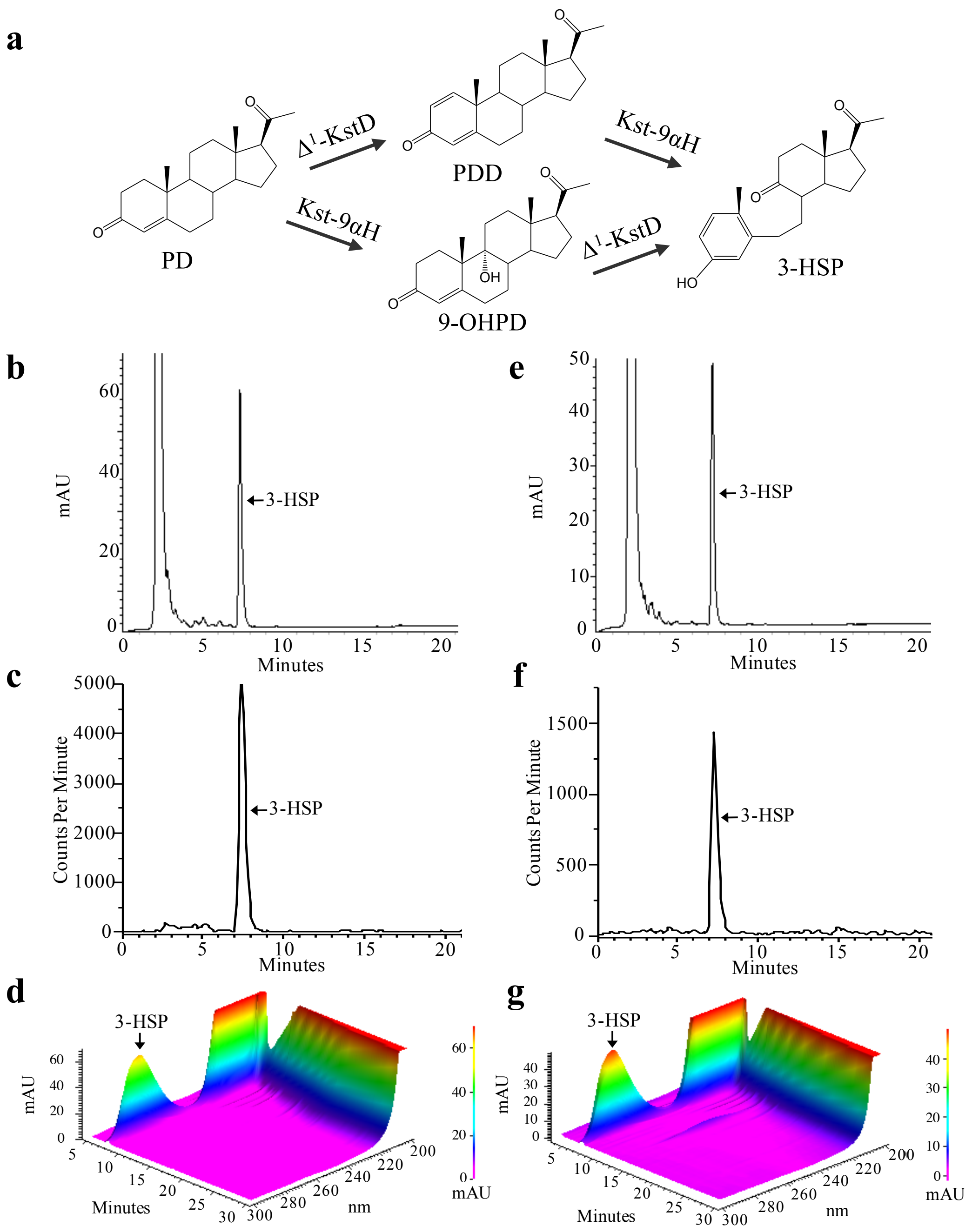

Figure S10. The combined activity of humanized 3-ketosteroid Δ^1-dehydrogenases (Δ^1-KstD$_R$ or Δ^1-KstD$_A$) with 3-ketosteroid-9α-hydroxylase (Kst-9αH) generates 3-hydroxy-9,10-secopregn-1,3,5(10)-triene-9,20-dione (3-HSP) from pregn-4-ene-3,20-dione (PD). Clarified lysates from *E. coli* expressing Δ^1-KstD$_R$ or Δ^1-KstD$_A$, and Kst-9αH were combined and incubated with C4-[^{14}C] labeled PD for 24 hours. Following incubation, lipids were extracted and analyzed by RP-HPLC as described in the methods. (a) Chemical structures and reaction overview. (b) Representative HPLC chromatogram of the 24 hour time point showing 3-HSP (λ_{max}: 280 nm; t_r = 7.2 min) in a combined sample from *E. coli* expressing Δ^1-KstD$_A$ and Kst-9αH. (c) [^{14}C] measured by in-line scintillation detector corresponding to the chromatogram shown in (b). (d) 3-D chromatogram showing the spectral data ($\lambda_{300\text{-}200\ nm}$) plotted against time and absorption (mAU) of the sample run in (b). (e) Representative HPLC chromatogram of the 24 hour time point showing 3-HSP (λ_{max}: 280 nm; t_r = 7.2 min) in a combined sample from *E. coli* expressing Δ^1-KstD$_R$ and Kst-9αH. (f) [^{14}C] measured by in-line scintillation detector corresponding to the chromatogram shown in (e). (g) 3-D chromatogram showing the spectral data ($\lambda_{300\text{-}200\ nm}$) plotted against time and absorption (mAU) of the sample run in (e).

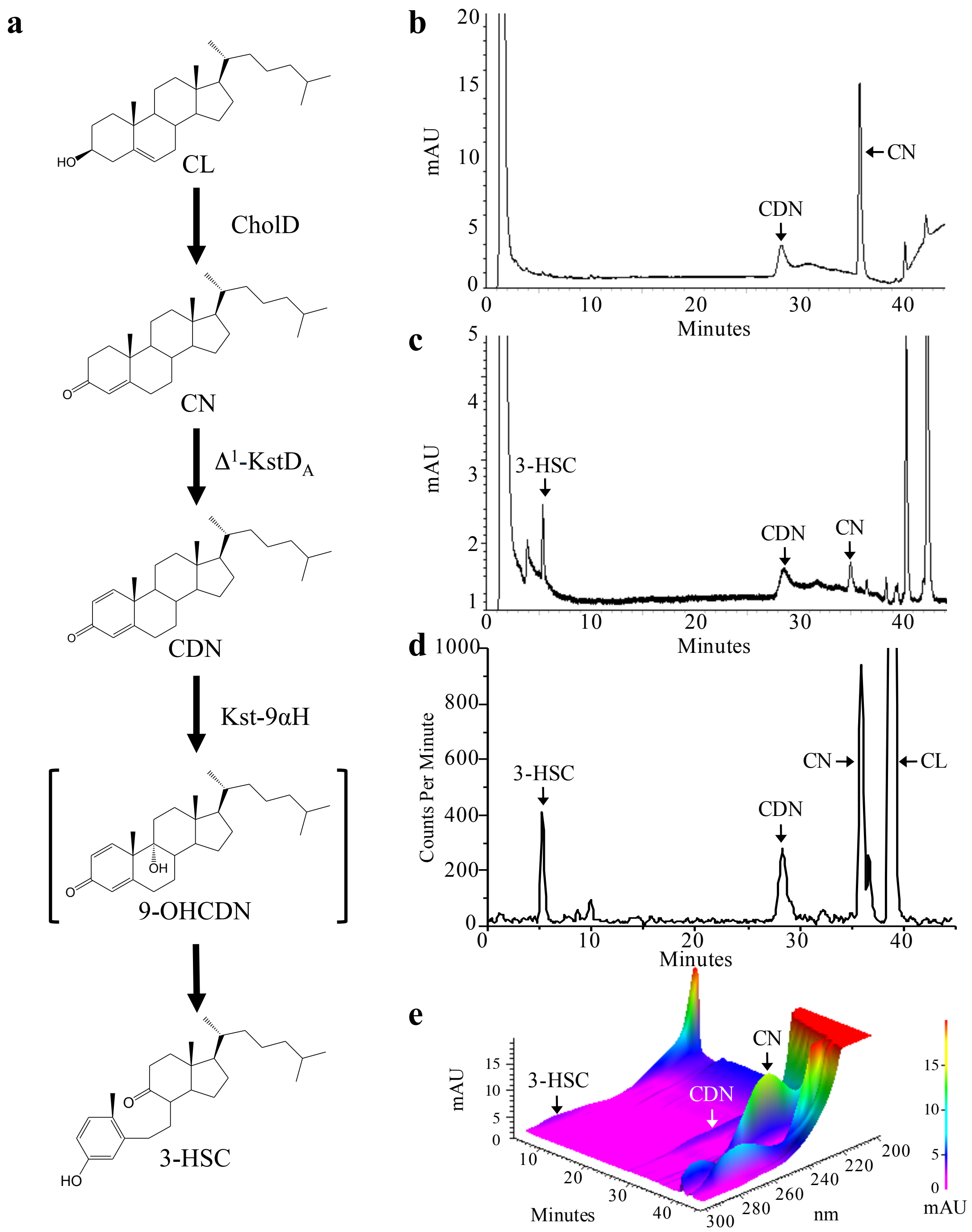

Figure S11. The combined activity of humanized cholesterol-3-OH dehydrogenase (CholD), 3-ketosteroid Δ^1–dehydrogenase (Δ^1-KstD$_A$), and 3-ketosteroid-9α-hydroxylase (Kst-9αH) generates 3-hydroxy-9,10-secocholestene-1,3,5(10)-triene-9-one (3-HSC) from cholesterol (CL). Clarified lysates from *E. coli* expressing CholD, Δ^1-KstD$_A$, or Kst-9αH were combined and incubated with C4-[^{14}C] labeled CL for 24 hours. Following incubation, lipids were extracted and analyzed by RP-HPLC as described in the methods. (a) Chemical structures and reaction overview. (b) Representative HPLC chromatogram at λ 239 nm and (c) λ 280 nm of the 24 hour time point showing cholestenone (CN; λ_{max}: 239 nm; t_r = 36.0 min), choleste-1,4-diene-3-one (CDN; λ_{max}: 241 nm; t_r = 29.5 min), and 3-HSC (λ_{max}: 280 nm; t_r = 5.3 min). (d) [^{14}C] measured by in-line scintillation detector corresponding to the chromatograms shown in (b, c). (e) 3-D chromatogram showing the spectral data ($\lambda_{300\text{-}200\ nm}$) plotted against time and absorption (mAU) of the sample run in (b, c).

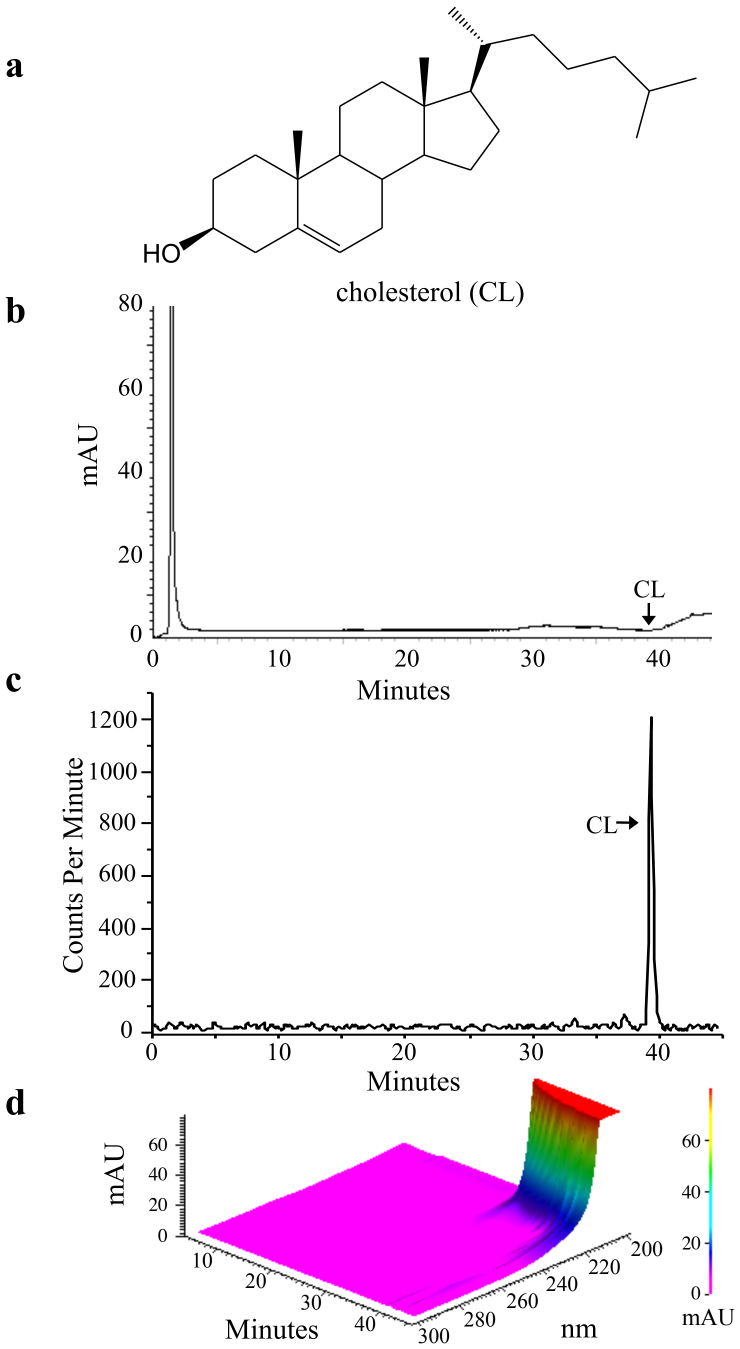

Figure S12. Clarified lysates produced from *E. coli* lack endogenous metabolic activity against cholesterol (CL). Clarified lysates from *E. coli* transformed with an empty (pUC19) expression plasmid (control) were incubated with C4-[^{14}C] CL for 24 hours. Following incubation, lipids were extracted and analyzed by RP-HPLC as described in the methods. (a) Chemical structure of CL. (b) Representative RP-HPLC chromatogram showing CL (λ_{max}: < 200 nm; t_r = 38.9 min) in a sample from *E. coli* transformed with an empty expression plasmid following 24 hours of incubation. (c) [^{14}C] measured by the in-line scintillation detector corresponding to the chromatogram show in (b). (d) 3-D chromatogram showing the spectral data ($\lambda_{300\text{-}200\ nm}$) plotted against time and absorption (mAU) of the sample run in (b).

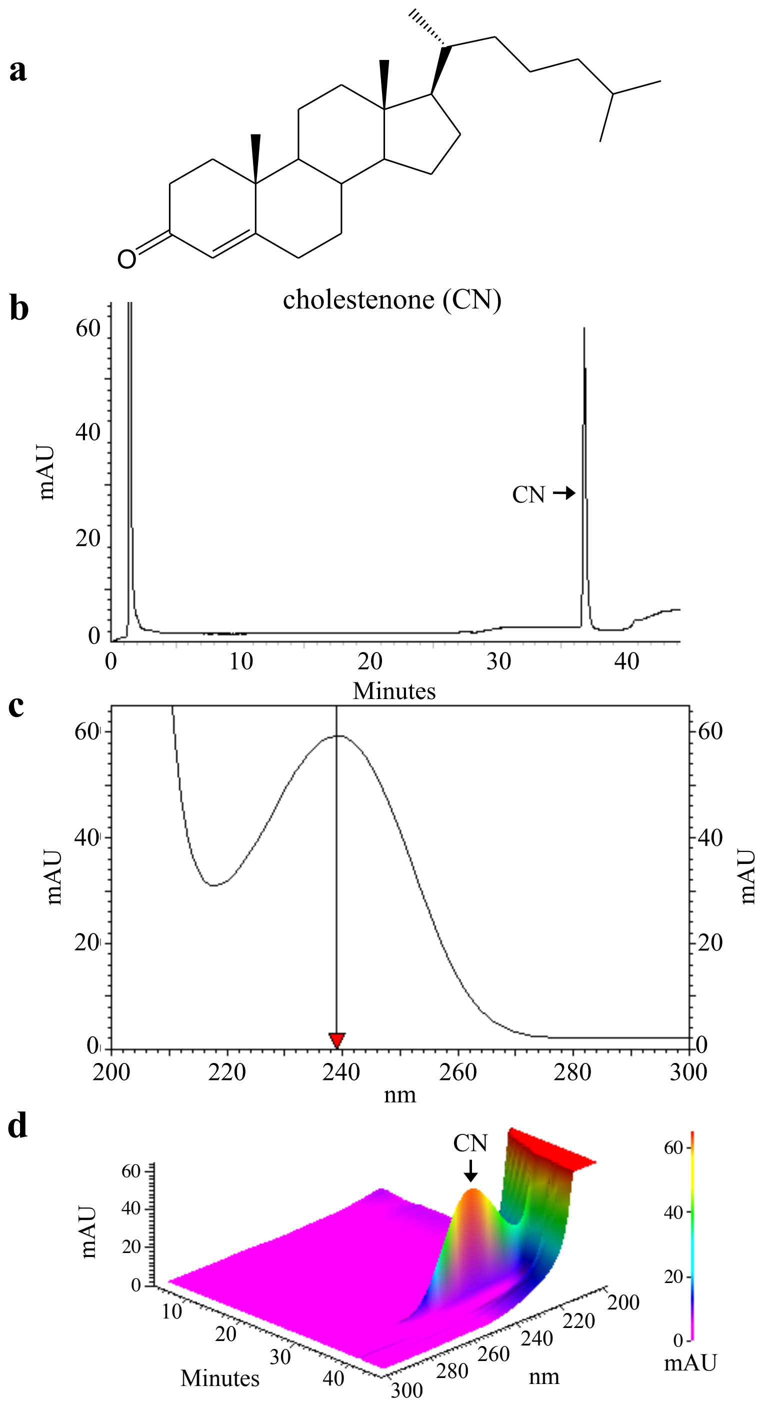

Figure S13. Clarified lysates produced from *E. coli* lack endogenous metabolic activity against cholestenone (CN). Clarified lysates from *E. coli* transformed with an empty (pUC19) expression plasmid (control) were incubated with CN for 24 hours. Following incubation, lipids were extracted and analyzed by RP-HPLC as described in the methods. (a) Chemical structure of CN. (b) Representative RP-HPLC chromatogram showing CN (λ_{max}: 239 nm; t_r = 36.9 min) in a sample produced from *E. coli* transformed with an empty expression plasmid following 24 hours of incubation. (c) UV absorption spectrum ($\lambda_{300\text{-}200\ nm}$) of CN in panel (b). (d) 3-D chromatogram showing the spectral data ($\lambda_{300\text{-}200\ nm}$) plotted against time and absorption (mAU) of the sample run in (b).

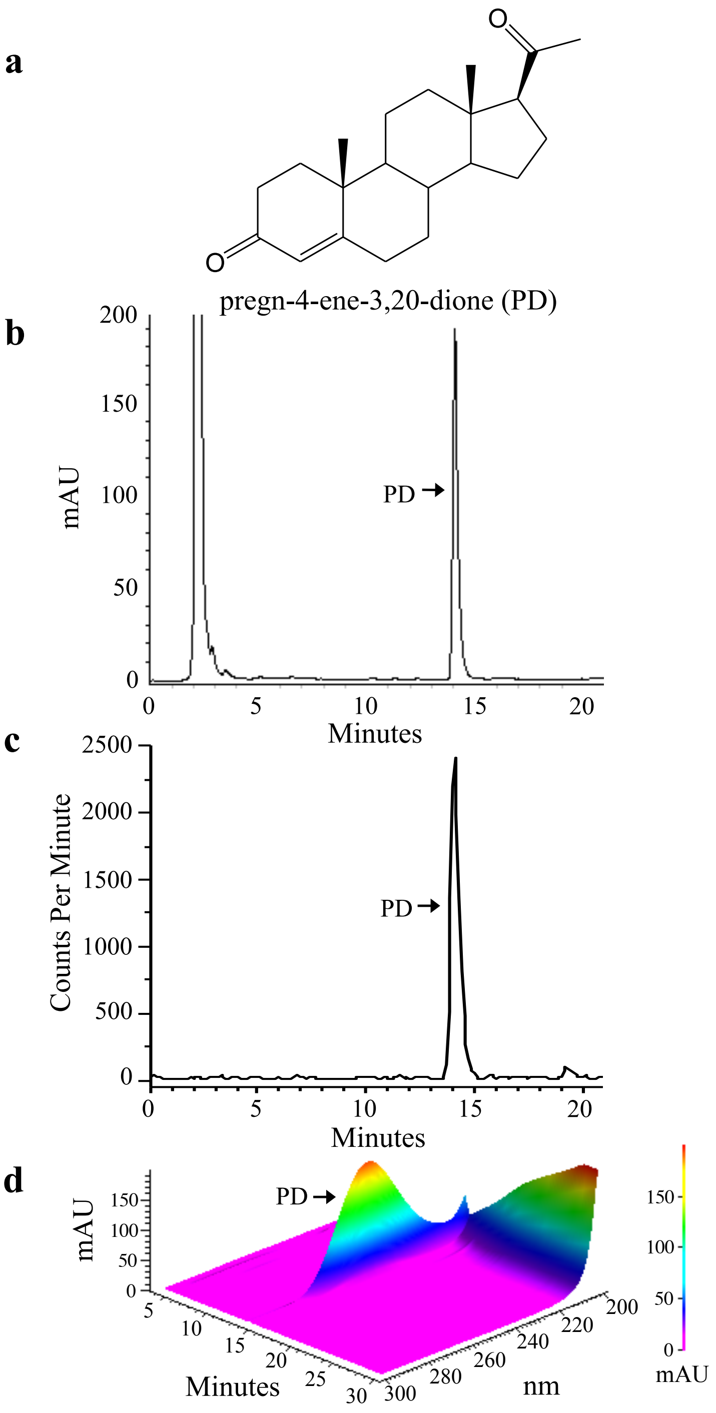

Figure S14. Clarified lysates produced from *E. coli* lack endogenous metabolic activity against pregn-4-ene-3,20-dione (PD). Clarified lysates from *E. coli* transformed with an empty (pUC19) expression plasmid (control) were incubated with C4-[^{14}C] PD for 24 hours. Following incubation, lipids were extracted and analyzed by RP-HPLC as described in the methods. (a) Chemical structure of PD. (b) Representative RP-HPLC chromatogram showing PD (λ_{max}: 245 nm; t_r = 13.8 min) in a sample from *E. coli* transformed with an empty expression plasmid following 24 hours of incubation. (c) [^{14}C] measured by the in-line scintillation detector corresponding to the chromatogram show in (b). (d) 3-D chromatogram showing the spectral data ($\lambda_{300\text{-}200\ nm}$) plotted against time and absorption (mAU) of the sample run in (b).

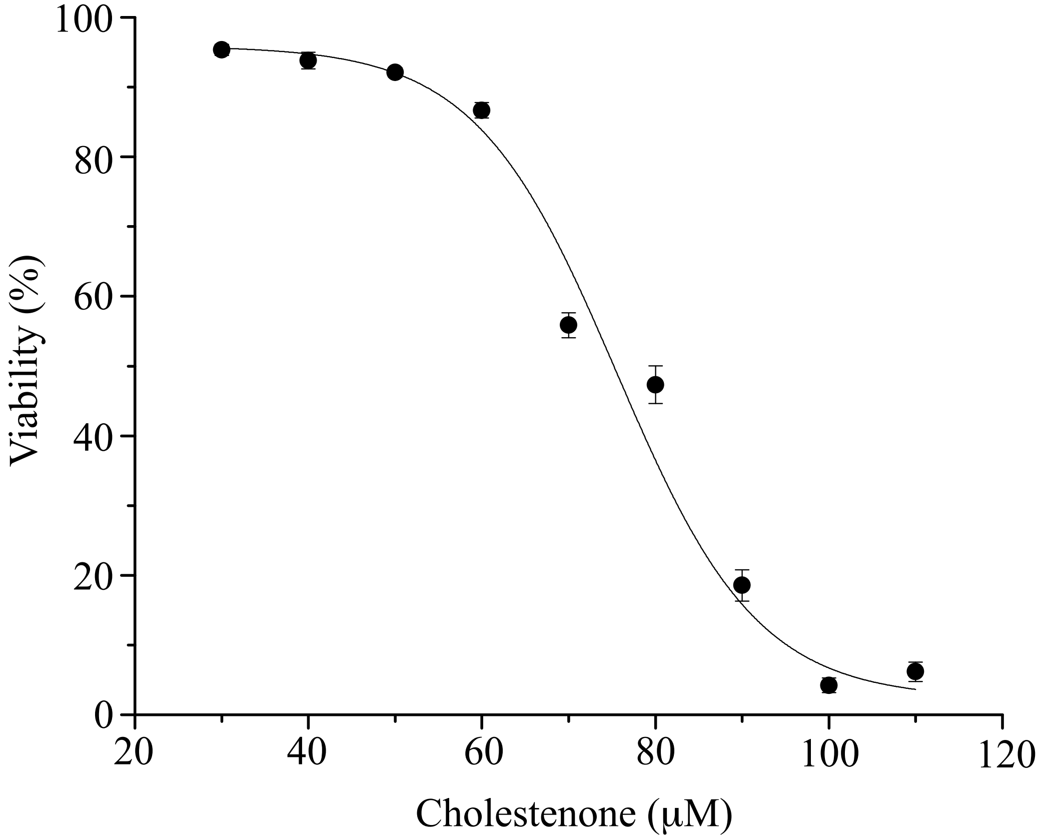

Figure S15. Cholestenone (cholest-4-ene-3-one) is toxic to human cells. Hep3B cells were seeded (6 x 10^4 cells/well) and allowed to grow until ~90% confluent. Cells were treated with cholestenone (30 - 110 μM) for 72 hours. After 48 hours of exposure, 120 μM of resazurin was added to each well for an additional 24 hours. Following incubation, the fluorescent intensity of each well was measured at 540 ± 25 ex and 620 ± 40 em with a BioTeK Synergy 2 plate reader. The mean fluorescent intensity from each concentration of cholestenone was plotted as a percent of the maximum fluorescent intensity. Data represents an N of 1 with 8 replicates. Error bars indicate the standard error of the mean.

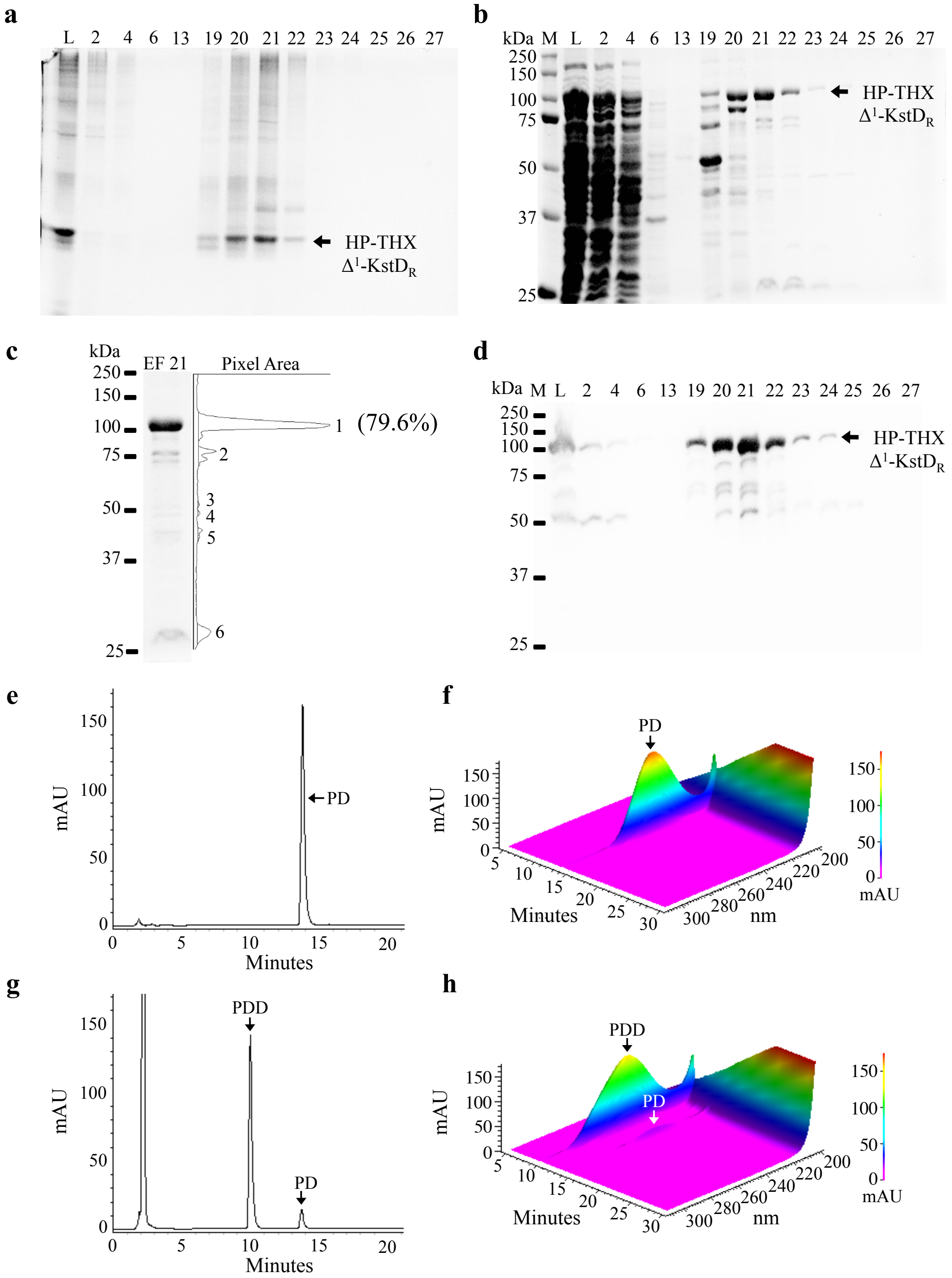

Figure S16 Expression, purification, and activity assessment of recombinant Δ^1-KstD$_R$. Recombinant Δ^1-KstD$_R$, expressed as FLAG-HP-THX-Δ^1-KstD$_R$ fusion protein in *E. coli* was partially purified using immobilized metal affinity chromatography (IMAC) and analyzed for purity and activity. (a) Image of a representative in-gel nitrotetrazolium blue (NTB) activity assay (Fig. S17) used to identify PD dehydrogenase activity in equivalent volumes of lysate (L) or the indicated elution fraction generated by IMAC after separation by Native-PAGE using a 10% polyacrylamide gel. (b) Coomassie blue stained 10% SDS-PAGE gel showing proteins contained in the lysate (L) and the indicated fractions generated during (IMAC) purification. (c) Cropped and enlargement of the image from elution fraction 21 of the Coomassie stained gel shown in (b) with an adjacent densitometry diagram generated with ImageJ used to assess the yield and purity of the Δ^1-KstD$_R$-fusion protein contained in the fractions. The concentration of protein in fraction 21 was determined to be 0.385 mg/mL with a purity of ~80%. (d) Western blot analysis from a replicate of the SDS-PAGE gel shown in (b) visualized with an anti-FLAG antibody. (e-h) RP-HPLC analysis showing PDD formation with a concomitant decrease in PD generated by the incubation of PD with partially (~80%) purified recombinant Δ^1-KstD$_R$. (e) Representative HPLC chromatogram showing PD (λ_{max}: 245 nm; t_r = 13.8 min) substrate. (f) 3-D chromatogram showing the spectral data ($\lambda_{300\text{-}200\ nm}$) plotted against time and absorbance of the sample run shown in panel (e). (g) Representative RP-HPLC chromatogram showing PDD (λ_{max}: 247 nm; t_r= 10.0 min) generated from PD (λ_{max}: 245 nm; t_r = 13.8 min) extracted from a reaction in which recombinant Δ^1-KstD$_R$ (770 ng) was incubated with (10 µM PD) for four hours at 37° C. (h) 3-D chromatogram showing the spectral data ($\lambda_{300\text{-}200\ nm}$) of the sample run shown in panel (g).

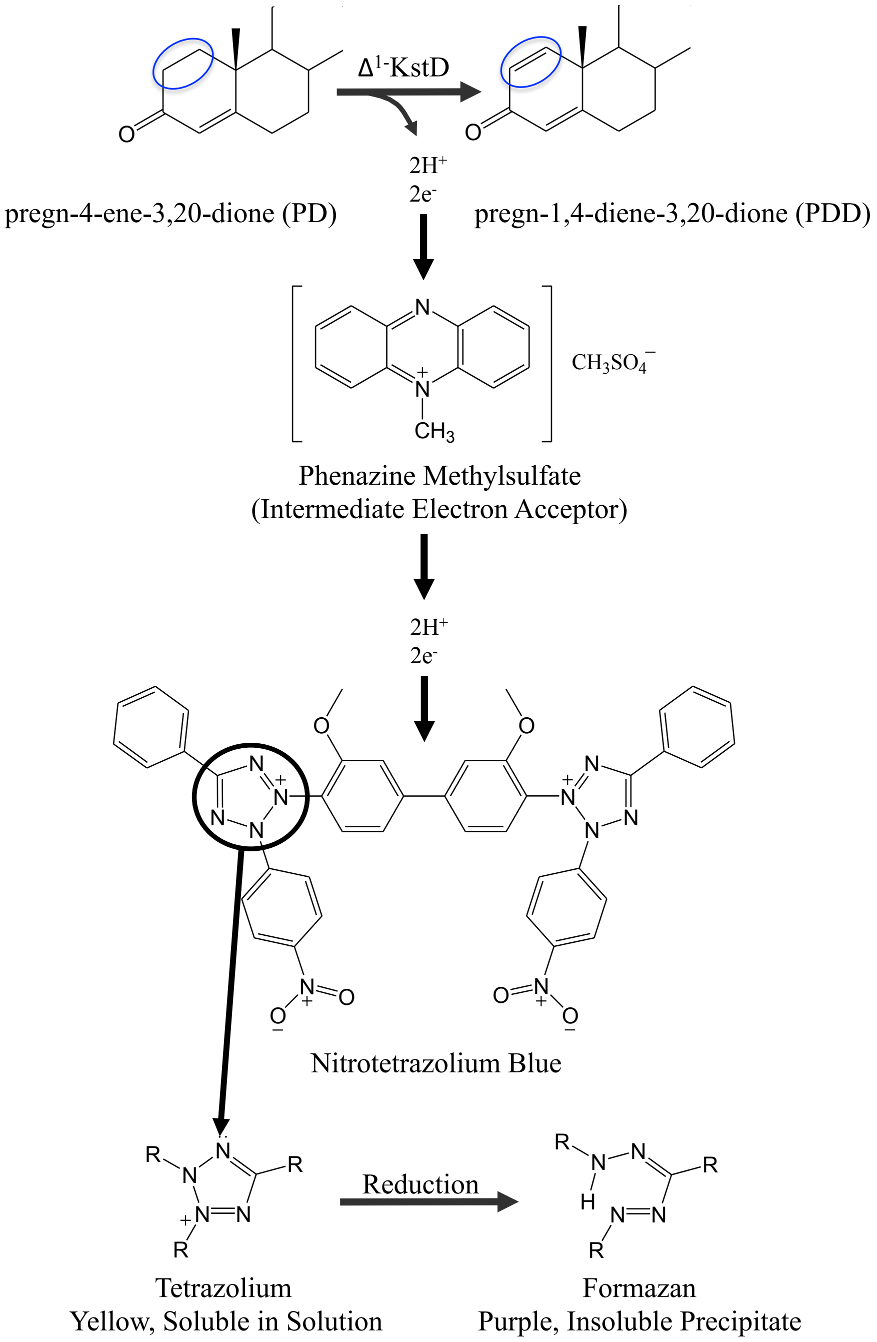

Figure S17. Nitrotetrazolium blue (NTB) reaction mechanism. The NTB assay is based of an indirectly coupled redox reaction that allows the assessment of the relative dehydrogenase activity within a sample. The reaction proceeds with the removal of two protons and two electrons from pregn-4-ene-3,20-dione (PD) by Δ^1-KstD to form pregn-1,4-diene-3,20-dione (PDD). The protons and electrons are donated from the FADH of Δ^1-KstD to the intermediate electron acceptor, phenazine methylsulfate (PMS). PMS relays the electrons to NTB where the center tetrazolium ring is reduced to formazan. The reaction causes NTB, which in the oxidized state forms a soluble yellow solution, to transition into an insoluble purple precipitate as dehydrogenation occurs. The assay was used in a native-PAGE format to assess the relative dehydrogenase activity from fractions produced during the purification of Δ^1-KstD$_R$ by immobilized metal affinity chromatography (IMAC) as described in the methods.

a

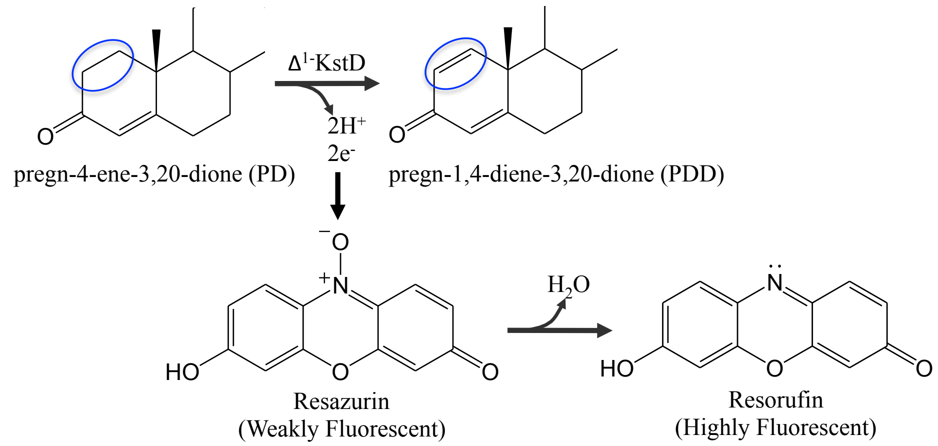

b

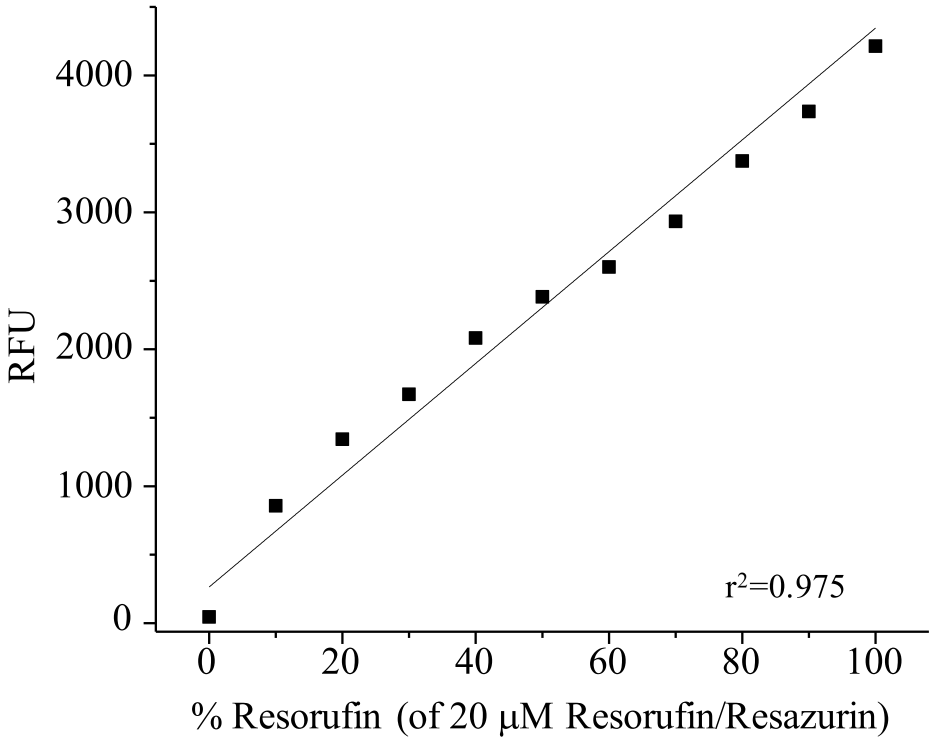

Figure S18. A resazurin-based fluorescent assay for kinetic measurements of Δ^1-KstD dehydrogenase activity. Resazurin, a weakly fluorescent redox dye, is irreversibly reduced upon accepting the two protons and two electrons released from the dehydrogenation of pregn-4-ene-3,20-dione (PD) producing pregn-1,4-diene-3,20-dione (PDD) by Δ^1-KstD. Upon reduction, resazurin forms the highly fluorescent compound, resorufin. (a) Structures and reaction summary. (b) Resorufin/resazurin standard curve. To best represent the reaction occurring in the resazurin enzyme assay during the dehydrogenation of pregn-4-ene-3,20-dione (PD) by Δ^1-KstD, inversely proportional concentrations of resazurin were added to resorufin. These values were expressed as a percent of resorufin, totaling 100% resorufin:resazurin. The standard curve shows a linear increase in fluorescence with increasing concentrations of resorufin in the presence of decreasing concentrations of resazurin.

a

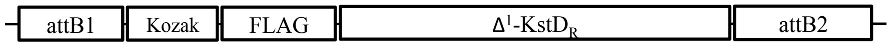

b

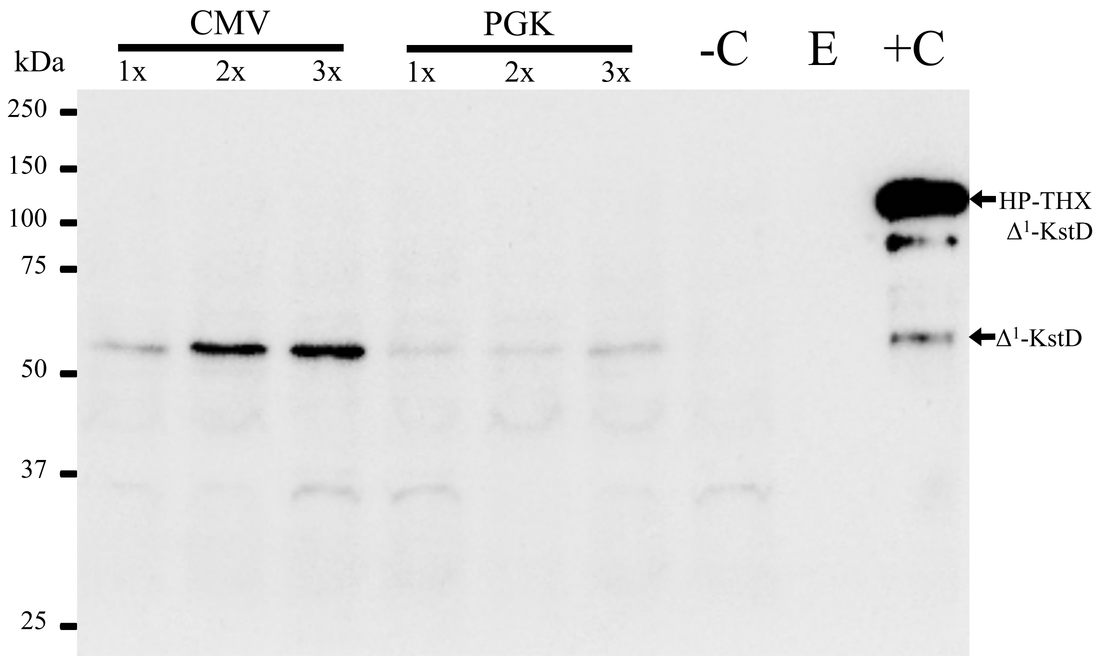

Figure S19. Stable expression of Δ^1-KstD$_R$ in Hep3B cells. Hep3B cells were transduced with increasing titers of lentiviral particles encoding Δ^1-KstD$_R$. Cells expressing Δ^1-KstD$_R$ were selected using blasticidin (CMV) or hygromycin (PGK) antibiotic as described in the methods. (a) Map of the eukaryotic Δ^1-KstD$_R$ expression construct encodes the humanized Δ^1-KstD$_R$ with a 5' Kozak consensus sequence for translation initiation. A FLAG tag was added to the N-terminal to aid in detection. Flanking attB attachment sites were added to aid subcloning via Gateway cloning. (b) Western blot analysis of Hep3B cells expressing CMV or PGK driven Δ^1-KstD$_R$. Samples include three representative CMV and PGK driven Δ^1-KstD$_R$ Hep3B cell lines, a negative control Hep3B lysate (-C), empty lane (E), and the isolated HP-THX-FLAG-Δ^1-KstD$_R$ as an anti-FLAG positive control (+C).

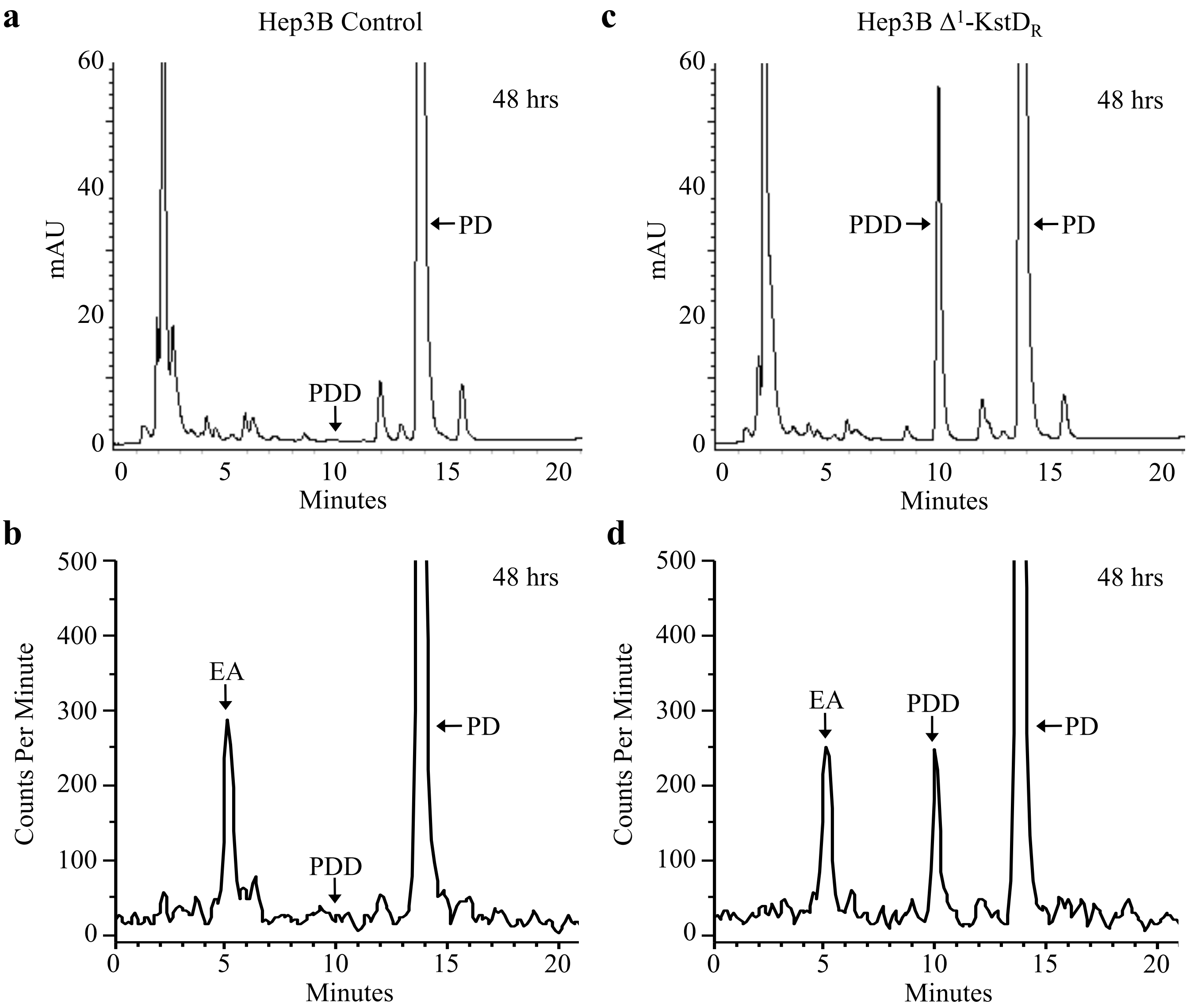

Figure S20. Δ^1-KstD$_R$ expression in Hep3B cells enables novel catabolic activity. Representative HPLC chromatograms (λ 245 nm) showing PD (t_r = 13.8 min) in controls (a,b) and Hep3B Δ^1-KstD$_R$ cells (c,d) after 48 hours showing the accumulation of PDD (λ_{max}: 247 nm; t_r = 10.0 min) only in the Hep3B cells expressing Δ^1-KstD$_R$ (c). (b) Representative image showing [^{14}C] trace detected by the in-line scintillation detector corresponding to the chromatogram shown in panel (a) revealing no PDD formation but a novel peak representing endogenous activity (EA; t_r = 5.2 min) in control cells. (d) [^{14}C] trace of the chromatogram shown in panel (c), showing PDD (t_r = 10.0 min) and the endogenous activity (EA; t_r = 5.2 min) with [^{14}C] present at a level that is comparable to that associated with PDD. For all experiments, Hep3B controls (left) or Hep3B Δ^1-KstD$_R$ cells (right) were incubated with 15.7 µg (10 µM) PD spiked with 100 nCi C4-[^{14}C] labeled PD (t_r = 13.8 min).

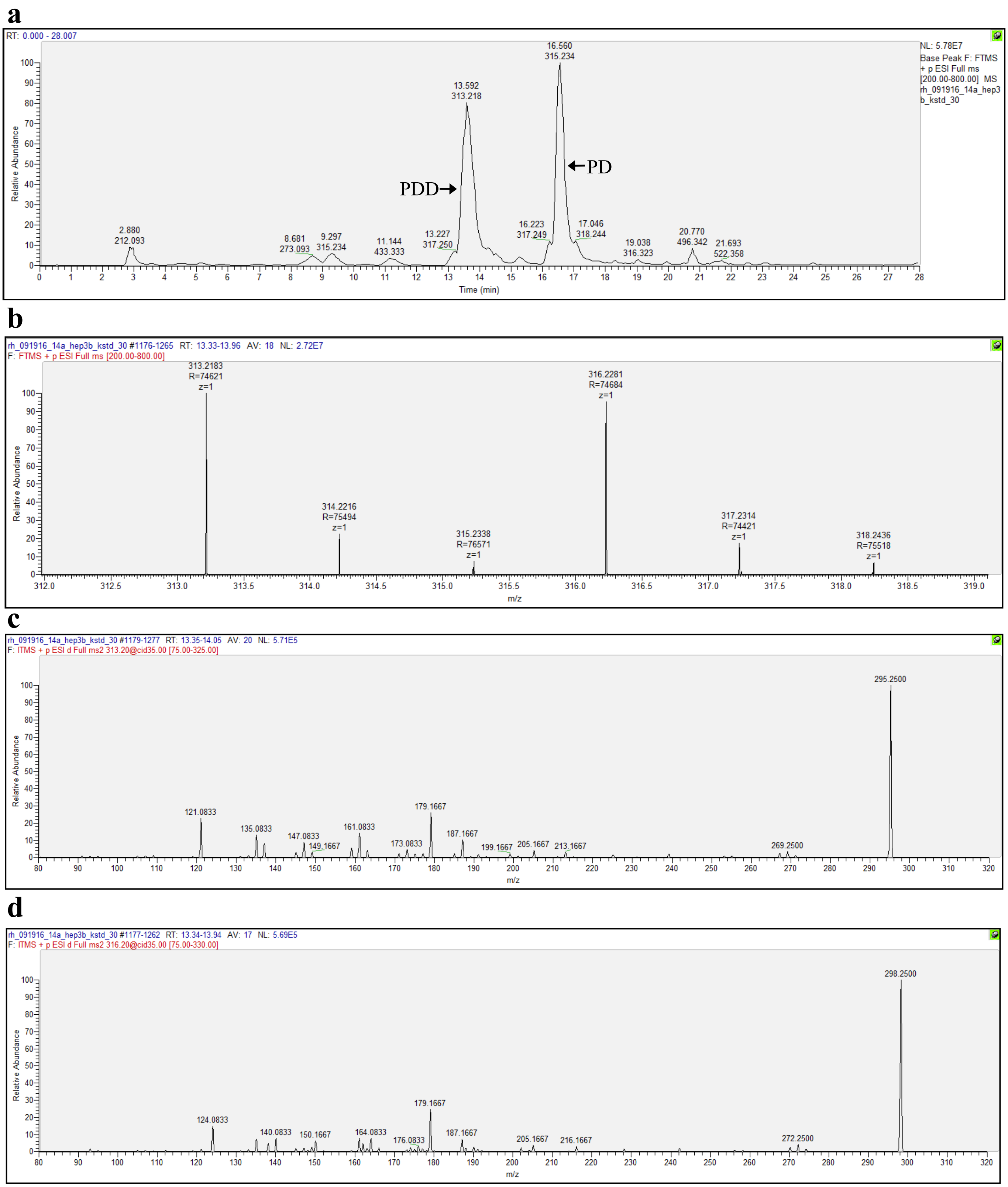

Figure S21. Mass spectrometry analysis confirming the production of pregn-1,4-diene-3,20-dione (PDD) from pregn-4-ene-3,20-dione (PD) C-1 and C-2 dehydrogenation by Hep3B cells expressing Δ^1-KstD$_R$. (a) Representative RP-HPLC chromatograms from Hep3B cells expressing Δ^1-KstD$_R$ following incubation with 5 μM PD and 5 μM C2,3,4-^{13}C-PD (t_r = 16.56 min) for 24 hours. (b) MS1 spectrum of PDD (t_r = 13.59 min) in panel (a) reveals two peaks representing unlabeled PDD (313.2183 m/z) and labeled PDD (316.2281 m/z). (c) MS2 spectrum of unlabeled PDD in panel (b). (d) MS2 spectrum of labeled PDD in panel (b).

a

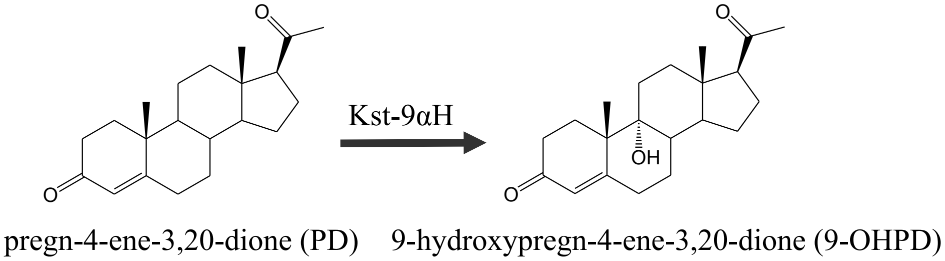

b

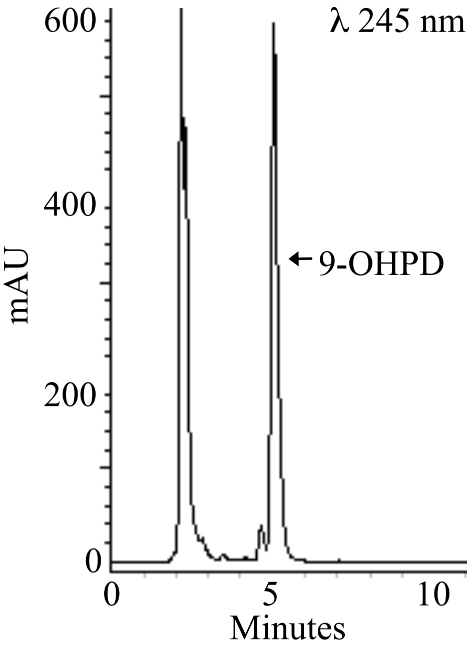

Figure S22. Generation of 9-hydroxypregn-4-ene-3,20-dione (9-OHPD). Clarified lysates produced from *E. coli* expressing 3-ketosteroid-9α-hydroxylase (Kst-9αH) were incubated with pregn-4-ene-3,20-dione (PD) for 48 hours. Following incubation, lipids were extracted and analyzed by RP-HPLC as described in the methods. (a) Structures and reaction summary. (b) Representative HPLC chromatogram of the 9-OHPD (λ_{max}: 245 nm; t_r = 5.2 min) product produced and isolated from clarified lysate.

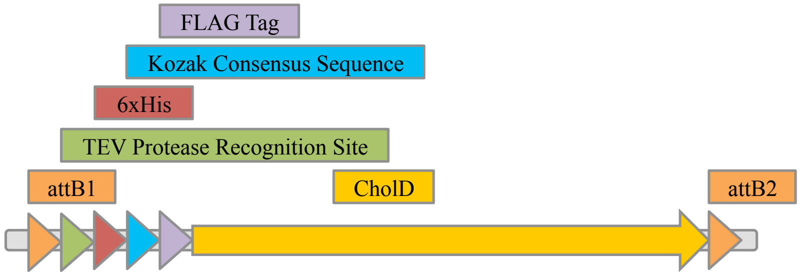

Figure S23. Overview of the humanized cholesterol-3-OH dehydrogenase (CholD) expression construct. The CholD expression construct encodes the CholD nucleotide sequence fused with a 5' Tobacco Etch Virus (TEV) protease recognition site, 6xHis tag to aid purification, a Kozak consensus sequence for translation initiation, and a FLAG tag to aid in detection. Flanking attB attachment sites were added to aid subcloning via Gateway cloning. Details are provided in Methods and the nucleotide sequence is provided in Table S1.

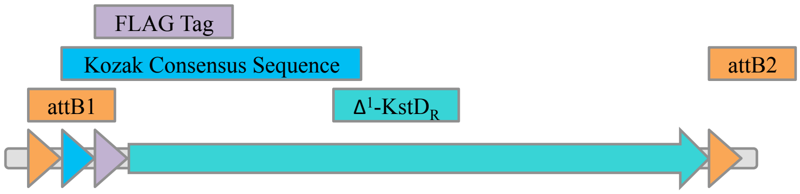

Figure S24. Overview of the humanized 3-ketosteroid Δ^1-dehydrogenase (Δ^1-$KstD_R$) expression construct. The Δ^1-$KstD_R$ expression construct encodes Δ^1-$KstD_R$ fused with a 5' Kozak consensus sequence for translation initiation. A FLAG tag was added to the N-terminal to aid in detection. Flanking attB attachment sites were added to aid subcloning via Gateway cloning. Details are provided in Methods and the nucleotide sequence is provided in Table S2.

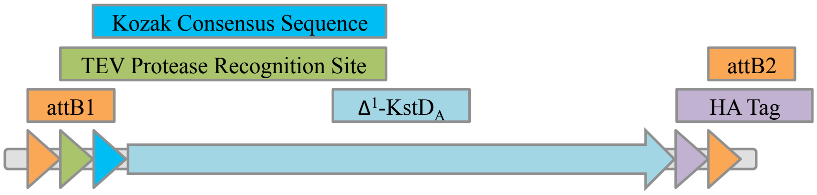

Figure S25. Overview of the humanized Δ^1-$KstD_A$ expression construct. The Δ^1-$KstD_A$ expression construct encodes the Δ^1-$KstD_A$ nucleotide sequence fused with a 5' Tobacco Etch Virus (TEV) protease recognition site, a Kozak consensus sequence for translation initiation, and a 3' HA tag to aid in detection when expressed in human cells. Flanking attB attachment sites were added to aid subcloning via Gateway cloning. Details are provided in Methods and the nucleotide sequence is provided in Table S3.

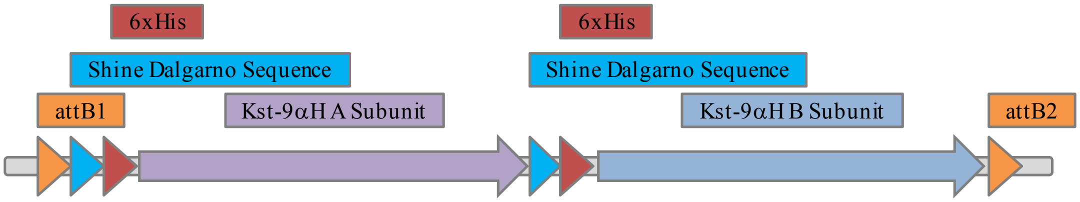

Figure S26. Overview of the 3-ketosteroid 9α-hydroxylase (Kst-9αH) expression construct used for expression in *E. coli*. The nucleotide sequence encoding the A (Kst-9αHA) and B (Kst-9αHB) subunits of Kst-9αH were fused with 5' Shine Dalgarno sequences for translation initiation, short linker peptides (amino acids: GAS), and 6x His tags to aid purification. The Kst-9αH expression construct was designed as a bicistronic construct by inserting a second shine dalgarno sequence following the 3' end of Kst-9αHA. The second Shine Dalgarno sequence was shifted by one nucleotide to produce a second open reading frame for coexpression of Kst-9αHB. Flanking attB attachment sites were added to aid subcloning via Gateway cloning. Details are provided in Methods and the nucleotide sequence is provided in Table S4.

Table S1. Cholesterol-3-OH dehydrogenase (CholD) nucleotide sequence

ACAAGTTTGTACAAAAAAGCAGGCTTCCAATTGGGATCCCTCGAGAACCTGTACTTCCAGGGCCACCACCACCACCACCACGGCACCATGGCCGGCTTCCTGA
ACTGCTGCCCCGGCTGCTGCATGGAGCCCGGCGCCAGCGACTACAAGGACGACGACGACAAGATGCTGCGGCGGATGGGAGATGCCAGCCTGACAACAGAGCT
GGGCCGGGTGCTCGTGACAGGCGGAGCTGGATTTGTGGGAGCCAACCTCGTGACCACCCTGCTGGATAGAGGACACTGGGTGCGCAGCTTCGACAGAGCCCCT
TCTCTGCTGCCTGCCCACCCTCAGCTGGAAGTGCTGCAGGGCGATATCACCGACGCCGATGTGTGTGCCGCCGCTGTGGATGGCATCGACACCATCTTTCACA
CCGCCGCCATCATCGAGCTGATGGGCGGAGCCAGCGTGACCGACGAGTACCGGCAGAGAAGCTTCGCCGTGAATGTGGGCGGCACCGAGAATCTGCTGCACGC
CGGACAGAGGGCTGGGGTGCAGAGATTCGTGTATACCAGCAGCAACAGCGTCGTGATGGGAGGCCAGAATATCGCTGGCGGCGACGAGACACTGCCCTACACC
GACAGATTCAACGACCTGTATACCGAGACAAAAGTGGTGGCCGAGCGCTTCGTGCTGGCCCAGAATGGCGTGGACGGCATGCTGACCTGCGCCATCAGACCTA
GCGGCATCTGGGGCAACGGCGACCAGACCATGTTCCGGAAGCTGTTCGAGAGCGTGCTGAAGGGCCACGTGAAGGTGCTCGTGGGCAGAAAGAGCGCCAGACT
GGACAACAGCTACGTGCACAACCTGATCCACGGCTTCATCCTGGCCGCTGCCCACCTGGTGCCTGATGGAACAGCTCCTGGACAGGCCTACTTCATCAACGAT
GCCGAGCCCATCAACATGTTCGAGTTCGCCAGACCCGTGCTGGAAGCTTGCGGCCAGAGATGGCCCAAGATGCGGATCTCTGGACCCGCCGTCAGATGGGTCA
TGACTGGCTGGCAGCGGCTGCACTTCAGATTCGGCTTTCCCGCCCCTCTGCTGGAACCCCTGGCTGTGGAAAGACTGTACCTGGACAACTACTTCTCTATCGC
CAAGGCCAGACGGGACCTGGGCTACGAGCCTCTGTTTACCACACAGCAGGCCCTGACCGAGTGCCTGCCCTACTACGTGTCCCTGTTCGAGCAGATGAAGAAC
GAGGCCAGAGCCGAGAAAACAGCCGCCACAGTGAAGCCCGGGTAGTAAACCGGTGAATTCAGATCTGACCCAGCTTTCTTGTACAAAGTGGT

*Sequences include flanking Gateway Technologies attachment (attB1 and attB2) sites for cloning into pEntr221.

**Details for cloning are provided in Methods.

Table S2. 3-ketosteroid Δ^1-dehydrogenase (Δ^1-$KstD_R$) nucleotide sequence

ACAAGTTTGTACAAAAAAGCAGGCTTCGAATTCAGATCTAGAAGTACTGGCACCATGGCCGGCGACTACAAGGACGACGACGATAAGATGCAGGACTGGACTA
GTGAATGCGACGTGCTGGTCGTGGGCTCTGGCGGAGGCGCTCTGACAGGCGCTTATACAGCTGCCGCCCAGGGCCTGACCACCATCGTGCTGGAAAAGACCGA
CAGATTCGGCGGCACCAGCGCCTACTCTGGCGCCTCTATTTGGCTGCCTGGCACCCAGGTGCAGGAAAGAGCCGGACTGCCTGACAGCACCGAGAACGCCAGA
ACCTACCTGAGAGCCCTGCTGGGCGACGCCGAGAGCGAAAGACAGGACGCCTACGTGGAAACCGCCCCTGCTGTGGTGGCTCTGCTGGAACAGAACCCCAACA
TCGAGTTCGAGTTCCGGGCCTTCCCCGACTACTACAAGGCCGAGGGCAGAATGGACACCGGCCGCAGCATCAACCCCCTGGATCTGGACCCTGCCGACATCGG
CGATCTGGCCGGAAAAGTGCGGCCCGAGCTGGACCAGGATAGAACCGGACAGGATCACGCCCCTGGCCCCATGATTGGAGGCAGAGCCCTGATCGGCAGACTG
CTGGCTGCTGTGCAGAGCACCGGAAAGGCCGAGCTGAGAACCGAGAGCGTGCTGACCAGCCTGATCGTGGAAGATGGCAGAGTCGTGGGCGCCGAGGTGGAAT
CTGGGGGCGAGACACAGCGGATCAAGGCCAACAGAGGCGTGCTGATGGCCGCTGGCGGCATCGAGGGAAACGCCGAGATGAGGGAACAGGCCGGAACACCCGG
CAAGGCCATCTGGTCTATGGGCCCCTTCGGAGCCAATACCGGCGACGCCATCTCTGCCGGAATTGCCGTGGGCGGAGCTACCGCACTGCTGGATCAGGCCTGG
TTCTGCCCTGGCGTGGAACAGCCTGATGGCAGCGCCGCCTTTATGGTGGGAGTGCGGGGAGGACTGGTGGTGGATTCTGCCGGGGAGAGATACCTGAACGAGA
GCCTGCCCTACGACCAGTTCGGCAGAGCTATGGACGCCCACGATGACAACGGCTCCGCCGTGCCCAGCTTCATGATCTTCGACAGCAGAGAGGGCGGAGGCCT
GCCCGCCATCTGCATCCCTAATACCGCCCCAGCCAAGCACCTGGAAGCCGGAACATGGGTGGGAGCCGACACACTGGAAGAACTGGCCGCCAAGACAGGCCTG
CCTGCCGATGCTCTGAGAAGCACCGTGGAAAAGTTCAACGACGCCGCCAAGCTGGGCGTGGACGAAGAGTTCCATAGAGGCGAGGACCCCTACGACGCCTTCT
TCTGCCCACCTAATGGCGGAGCCAACGCCGCCCTGACCGCCATTGAGAACGGCCCTTTTTACGCCGCCAGAATCGTGCTGAGCGACCTGGGCACAAAGGGCGG
CCTCGTGACCGATGTGAACGGCAGAGTGCTGAGAGCCGACGGCAGCGCCATTGACGGACTGTATGCCGCCGGAAATACCAGCGCCAGCCTGAGCGGCAGATTC
TACCCTGGCCCAGGCGTGCCACTGGGCACCGCTATGGTGTTCAGCTACAGAGCTGCCCAGGACATGGCGAAGTAATTCTAGAGAGCTCAAGGTGAATTCAGAT
CTGACCCAGCTTTCTTGTACAAAGTGGT

*Sequences include flanking Gateway Technologies attachment (attB1 and attB2) sites for cloning into pEntr221.

**Details for cloning are provided in Methods.

Table S3. 3-ketosteroid Δ^1-dehydrogenase (Δ^1-$KstD_A$) nucleotide sequence

ACAAGTTTGTACAAAAAAGCAGGCTTCCAATTGGGATCCGGTACCGAAAACCTGTACTTCCAGGGTACCATGAGCATCGAGACAAACACCTACGACGTGATCG
TCGTGGGCTCTGGCGCTGGCGCTATGCTGGCTGCTGCTAGAGCCCATGATCTGGGCCTGAGCGTGCTGGTGGTGGAAAAGAGCGATAAGTACGGCGGCACCAG
CGCCGTGTCTGGCGGAGCTGTGTGGATTCCCAACAACAGCCAGATGCAGATCAAGGACAGCTTCGACGAGGCCCTGACCTACCTGAAGGCCGCCACACAGGGA
CTGGTGGCCGAGGATAGACTGCTGGCCTACCTGGAAAGCGCCCCTCAGATGGTGGAGTACATCAACGCCAATATGACCCTGCAGTACTTCCCCTGCCACAGAT
ACCCCGACTACTACCAGCATCTGCCTGGCGCCAAGCCTGGCGGCAGAACCATGGAACCCATGCTGTTCGATGCCGCCCTGCTGGGCGACGAGTTCGCCAATCT
GAGAATGGCCTACACCGGCACCCTGCTGATGGGCAAGGCCAGCATGACAGCCACAGAGGCCCATGTGATGCTGGCCAAAGAACCCGGCTGGATGCTGCAAGTG
ATCAAGAGCCTGGGCCGGTACTACCTGGACCTGCCCTGGCGGCTGAAGTCCCGGCACGATAGAAAGAGAGGCCTGGGCAACGCCATGGCCGCTGGACTGAGAC
ACGCTCTGCTGGAAAGAAAGGTGCCCCTGTGGCTGAACACCCCCTTCGAGAGCCTGATCACAGAGGGCGCCGAGAACAAGCGCGTGACCGGCATCGTCGTGAA
GCGGAATGGCCAGACACTGCAGCTGACCGCCAGACGGGGAGTGGTGCTGGGAGCTGGCGGCTTCGAGAGAAACCAGCAGATGAGAGAGCAGTACCTGCCCAAG
CCCACCAACGCCGCTTGGAGCGCTACCCCCCCTCACAATACCGGCGACACAATCAGAGCCGCCATGGACATCGGAGCCAGAGCCGAGCTGATGGACTGGGCTT
GGTGGGTGCCATCCATCCACGTGCCAGGCGAAGCCGCTCAGACCGGACTGTTCGCCGAGAGAAATCTGCCCGGCTGCATCGTCGTGAATGGCAAGGGCCAGCG
GTTCATCAACGAGGCCAGCCCTTACCTGGAATTTGGCGCCGCTATGTACGAGAACCACGCCAGATCCGGCTCTGCCGTGCCTGCCTGGCTGATCTTCGACGGC
AAGTTCCGGTACAACTACCCCATGGGCCCCCTGATGCCTGGCCAGATCCAGCCTGATAGAAAGGCCTGGCTGGGCAAGGTGTACTGGCGGGACGATACACTGG
AAGGACTGGCCAAGCAGATCGGCGTGGACGCTGCCGGACTGAAGCAGTCCGTGGAACTGAACAACCAGTACGCCCAGGACGGCAAGGACAGAGAGTTCGACAA
GGGCGGCAACGTGTTCGATCGGTACTACGGCGACTACAACGTGAAGCCCAACCCTTGCCTGGCCCCCATCGGCAAGCCTCCCTACTACGCCATGAGAGTGGAC
GCCGGGGACATCGGCACAAAGGGCGGACTGCTGACCGACAAGGACGCCAGAGTGCTGGACGAGAGCGACAGACCTATCGAGGGCCTGTACTGCATCGGCAACA
ACTCCGCCAGCGTGATGGGAAAAGCCTACCCTGGCGCAGGCGGCACACTGGGACCTGCCATGACCTTCGGCTTTAGGGCCGCCAACCACATTGCCGCCAGCAA
GTACCCCTACGATGTGCCCGATTACGCCGGCTAGTAACCCGGGGAATTCAGATCTGACCCAGCTTTCTTGTACAAAGTGGT

*Sequences include flanking Gateway Technologies attachment (attB1 and attB2) sites for cloning into pEntr221.

**Details for cloning are provided in Methods.

Table S4. 3-ketosteroid-9α-hydroxylase (Kst-9αH) nucleotide sequence

ACAAGTTTGTACAAAAAAGCAGGCTTCGGATCCTAAGGAGGTAACATCTATGGGTTATGGTCGTAAAAAACGTCGTCAGCGTCGTCGTGGTGCAAGCCATCAT
CATCACCATCATGGTAGCATGAGCATTGATACCGCACGTAGCGGTTCAGATGATGACGTTGAAATTCGTGAAATTCAGGCAGCAGCAGCACCGACCCGTTTTG
CACGTGGTTGGCATTGTCTGGGTCTGCTGCGTGATTTTCAGGATGGTAAACCGCATAGCATTGAAGCATTTGGCACCAAACTGGTTGTTTTTGCAGATAGCAA
AGGTCAGCTGAATGTTCTGGATGCATATTGTCGTCATATGGGTGGTGATCTGAGCCGTGGTGAAGTTAAAGGTGATAGCATTGCATGTCCGTTTCATGATTGG
CGTTGGAATGGTAAAGGTAAATGTACCGATATTCCGTATGCACGTCGTGTTCCGCCTATTGCCAAAACCCGTGCATGGACCACCCTGGAACGTAATGGCCAGC
TGTATGTTTGGAATGATCCGCAGGGTAATCCGCCTCCGGAAGATGTGACCATTCCGGAAATTGCAGGTTATGGCACCGATGAATGGACCGATTGGAGCTGGAA
AAGCCTGCGTATTAAAGGTAGCCATTGCCGTGAAATTGTTGATAATGTTGTTGATATGGCCCACTTCTTCTATATCCATTATAGCTTTCCGCGTTATTTCAAA
AACGTGTTTGAGGGTCATACCGCAACCCAGTATATGCATAGCACCGGTCGTGAAGATGTTATTAGCGGCACCAATTATGATGATCCGAATGCAGAACTGCGTA
GCGAAGCAACCTATTTTGGTCCGAGCTATATGATTGATTGGCTGGAAAGTGATGCAAATGGCCAGACCATTGAAACCATTCTGATTAATTGTCATTACCCGGT
GAGCAACAATGAGTTTGTTCTGCAATATGGTGCCATCGTGAAAAAACTGCCTGGTGTTAGTGATGAAATTGCCGCAGGTATGGCAGAACAGTTTGCAGAAGGT
GTTCAGCTGGGTTTTGAACAGGATGTTGAAATTTGGAAAAACAAAGCACCGATTGATAATCCGCTGCTGAGCGAAGAAGATGGTCCGGTTTATCAGCTGCGTC
GTTGGTATCAGCAGTTTTATGTTGATGTGGAAGATATCACCGAGGATATGACCAAACGCTTTGAATTTGAAATTGATACCACCCGTGCAGTTGCAAGCTGGCA
GAAAGAAGTTGCAGAAAATCTGGCAAAACAGGCAGAAGGTAGCACCGCCACCCCGTAAGAATTCTAAGGAGGTAACATCTATGGGCTACGGACGCAAAAAACG
CCGTCAACGGCGTCGCGGAGCAAGTCACCACCACCATCACCATGGTAGTATGACCGCAGTTCAGGCACCGGTTACCAGCCGTGCAACCGTTCTGACCGTTAGC
GCAGTTGTTCAAGAAACCGCAGATGCAGTTAGCCTGGTTTTTGATGTTCCGGATGATCGTCGCGAAGATTTTACCTATCGTCCGGGTCAGTTTCTGACCCTGC
GCATTCCGAGCGATCGTACCGGTAGCGTTGCACGTTGTTATAGCCTGGCAAGCAGCCCGTTTACCGGTGAACCGCCTAAAGTTACCGTTAAACGTACCGCAGG
CGGTTATGGTAGCAATTGGCTGTGTGATAATATTGTGGCAGGTCGTAGTATTGAAGTTCTGCCTCCGGCAGGCGTTTTTACACCGGCAGATCTGACCGAAAAA
CTGGTTCTGTTTGCCGGTGGTAGCGGTATTACACCGGTTATGAGCATTCTGGAAAGCGCACTGCATAGCGGTAATCGTGATGTTGTTCTGATTTATGGTAATC
GCGACGAAAAAAGCGTTATCTTTGCGGAAAAACTGCGTGAACTGGCAGCACGTCATGCCGGTGCACTGACCGTTGTTCACTGGCTGGAATCAGTTCAGGGTCT
GCCGAGTCCGCAGCAGCTGGCAACCCTGATTAGCCCGTTTGCAGATCATCGTGCATATATGTGTGGTCCGGGTCCTTTTATGGATACCGTTCGTGAAGGCCTG
CTGCTGGCTGGTGTTCCGAAAGATCGTATTCATGCAGAAGTTTTTACCAGCCTGAGCGGTGATCCGTTTGCCGATGTTCCGCTGGTTGAAATCGATGAATCAG
ATGCAGATGCAACCAGCGCCACCGTGCAGCTGGATGGTGAAGAACATGATCTGGTTTGGCCTCGTAGCGCAACCCTGGTTGATGTTATGCTGAGTAAAGGTCT
GGATGTTCCGTATAGCTGTCGTGAAGGTGAATGTGGTAGCTGTGCGTGTACCGTTGTTGAAGGTGATGTTGATAGCCTGCCGAGCGCAATTCTGGATGAAGAA
GATATTGCAAACGGTTATGTTCTGGCATGTCAGGCACGTCCGAAAAGCGATCATGTGCGTATTGAATTTTGATAACTGCAGGACCCAGCTTTCTTGTACAAAG
TGGT

*Sequences include flanking Gateway Technologies attachment (attB1 and attB2) sites for cloning into pEntr221.

**Details for cloning are provided in Methods.
